# Spatiotemporal control of immunogenic cell death: rewiring tumor-immune dialogues for next-generation immunotherapy

**DOI:** 10.3389/fimmu.2026.1784935

**Published:** 2026-03-03

**Authors:** Chengbin Li, Lu Zhu, Yubing Wang, Lei Zhao, Xing Lin, Zhixian Sun, Tingxi Yan, Yingduo Wang, Junjie Piao, Aihua Jin

**Affiliations:** 1 Department of Gastroenterology, Affiliated Hospital of Yanbian University, Yanji, China; 2 Key laboratory of Pathobiology (Yanbian University), State Ethnic Affairs Commission, Yanji, China; 3 Department of Thoracic Surgery, Yanbian University Hospital, Yanji, China

**Keywords:** cancer, DAMPs, immunogenic cell death, immunotherapy, TME

## Abstract

Immunogenic cell death (ICD) is a regulated cell death process distinguished by its ability to stimulate an adaptive immune response. This occurs through the emission of damage-associated molecular patterns (DAMPs), such as calreticulin (CRT), adenosine triphosphate (ATP), High Mobility Group Box 1 (HMGB1), type I interferons (IFN-α/β), and heat shock proteins(HSPs). Collectively, these signals promote dendritic cells (DCs) maturation, facilitate antigen cross-presentation, and trigger cytotoxic T lymphocytes (CTLs) activation. This cascade of immunostimulatory events is critical for converting immunologically “cold” tumors into “hot” ones. This review systematically explains the molecular mechanism of ICD, focusing on the space-time regulation of DAMPs emission and their role in remodeling the tumor immune environment. We also list a variety of ICD inducers, including conventional chemotherapeutic drugs, targeted drugs, nanotechnology-driven systems, physical means, and tumor-lytic viruses. The core theme is the synergistic potential of ICD with immune checkpoint inhibitors(ICIs), chimeric antigen receptor T cells (CAR-T cells)therapy, and microbiome regulation, supported by emerging preclinical and clinical evidence. We also discuss some current challenges, such as the heterogeneity of tumors released by DAMPs and immune escape mechanisms, and explore the development of biomarkers for patient stratification. In the future, we have emphasized some promising research directions, including artificial intelligence-assisted drug design, spatially differentiated metometric technology, and engineered immune cell therapy to achieve precise space-time-induced immune cell death. This review presents the mechanistic insights and transformative research directions for positioning ICD as a central pillar in the future landscape of immuno-oncology.

## 1 Introduction

**Figure 1:**
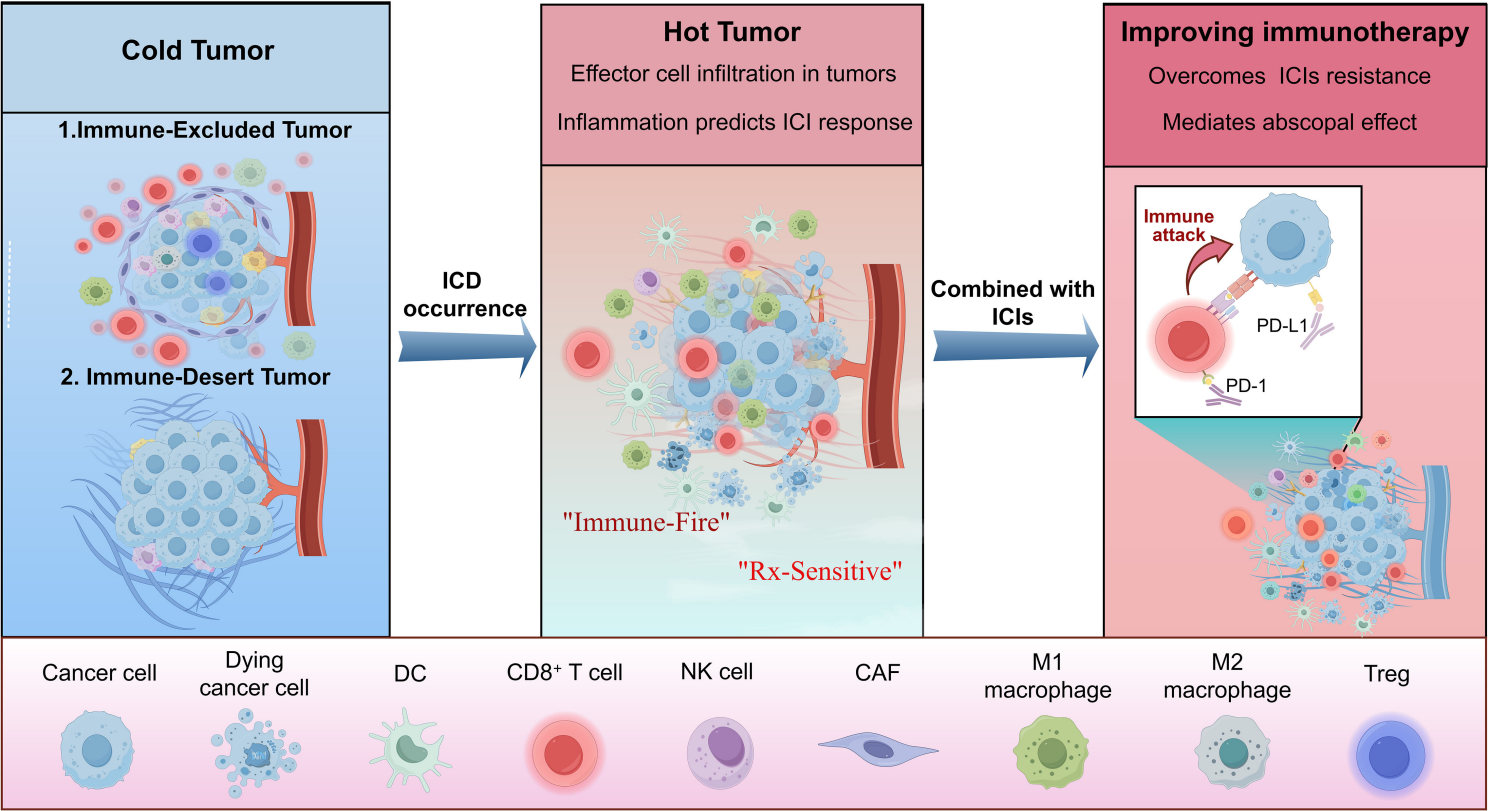
Illustrates the contrasting profiles of cold versus hot tumors and how ICD influences the tumor immune microenvironment. Immunologically “cold” tumors typically demonstrate minimal immune cell infiltration and a predominance of immunosuppressive cells; these are categorized as either immune-excluded or immune-desert phenotypes. Conversely, “hot” tumors are distinguished by substantial infiltration of effector immune cells, including CD8+ T cells and NK cells, throughout the tumor core and its margins. This immunologically active state often predicts a favorable response to ICIs. A crucial process for shifting a cold tumor to a hot phenotype is ICD, which promotes the recruitment and functional activity of tumor-infiltrating lymphocytes. The efficacy of ICIs can be significantly augmented by this ICD-driven transformation, ultimately leading to enhanced anti-tumor immunity and improved treatment outcomes.

Recent progress in cancer immunology has revealed the key role of the immune system in controlling the occurrence and development of tumors, thus giving rise to the concept of “cancer immune editing.”This framework describes three dynamic stages of tumor cell interactions (1, 2): immune elimination, balance, and escape. In the elimination stage, the innate immune system identifies tumor-related antigens through pattern recognition receptors (PRRs) and works with adaptive immunity to clear tumor cells. In the equilibrium stage, tumor cells are not completely eradicated but are controlled by effective cells, such as CTLs and natural killer cells (NKcells). Finally, in the escape stage, the tumor escapes immune surveillance through a variety of strategies, such as modulation of major histocompatibility complex (MHC) molecules, upregulation of immune checkpoint molecules, such as programmed death ligand 1 (PD-L1), and culture of immunosuppressive ecological positions. Tumors are usually classified according to their immune environment: due to insufficient T cell infiltration and inactivity of interferon γ (IFN-γ)signals, “cold tumors” have a poor response to immunotherapy such as immune checkpoint inhibitors (ICIs), while “hot tumors” usually respond better, thanks to a large number of T cell infiltration and pro-inflammatory cytokine secretion (3, 4). Therefore, the core challenge in tumor immunotherapy is designing immunomodulation strategies to effectively convert cold tumors into hot tumors (**Figure 1**).

Therefore, ICD has become a new treatment that can reprogram the immune microenvironment of tumors. Unlike other forms of cell death, ICD causes endoplasmic reticulum (ER) stress, mitochondrial dysfunction, and accumulation of reactive oxygen (ROS), eventually leading to the release of DAMPs (5). These include surface-exposed CRT, secreted ATP, and passively released HMGB1. These DAMPs bind to PRRs on DCs, such as TLR4, CD91, and p2rx7, thus promoting the cross-presentation of tumor antigens and enhancing anti-tumor immunity mediated by CD8+ T cells. ICD not only improves the immunogenicity of tumor cells but also promotes the development of tertiary lymphatic structures (TLS) and improves the tumor immune landscape by collecting CXCR3+ T cells (6). At present, a range of anticancer approaches employed in clinical settings, including anthracycline-based chemotherapeutic agents, oxaliplatin, radiation therapy, and emerging modalities, such as photodynamic therapy and oncolytic virotherapy, have demonstrated the capacity to trigger ICD. These strategies, particularly when combined with other treatments, such as immune checkpoint blockade (ICB), exhibit synergistic effects in preclinical models. Consequently, a deeper investigation into the molecular mechanisms underlying ICD, along with its integrative use with established immunotherapeutic regimens, is of substantial theoretical and clinical relevance. Such efforts are crucial for advancing novel anticancer treatments and addressing the challenges related to tumor immunotherapy resistance.

In this study, we build on and extend previous authoritative reviews on ICD and regulated cell deathin several ways. First, we provide a more systematic overview of the spatiotemporal regulation of DAMPs emission, emphasizing how the timing and anatomical context of DAMPs exposure or release shape downstream immunological outcomes. Second, we offer an in-depth discussion of emerging regulated cell death modalities and their crosstalk, highlighting mechanistic intersections and their potential implications for ICD-based therapeutic strategies. Third, we catalogue current ICD-inducing approaches and rational combination regimens-including chemotherapy, targeted agents, and physical modalities-in greater detail, and discuss how these interventions can be integrated to enhance antitumor immunity. Collectively, these elements distinguish this review from previous ones and provide a refined framework to guide future translational studies.

## 2 Hallmarks of ICD: DAMPs

**Figure 2:**
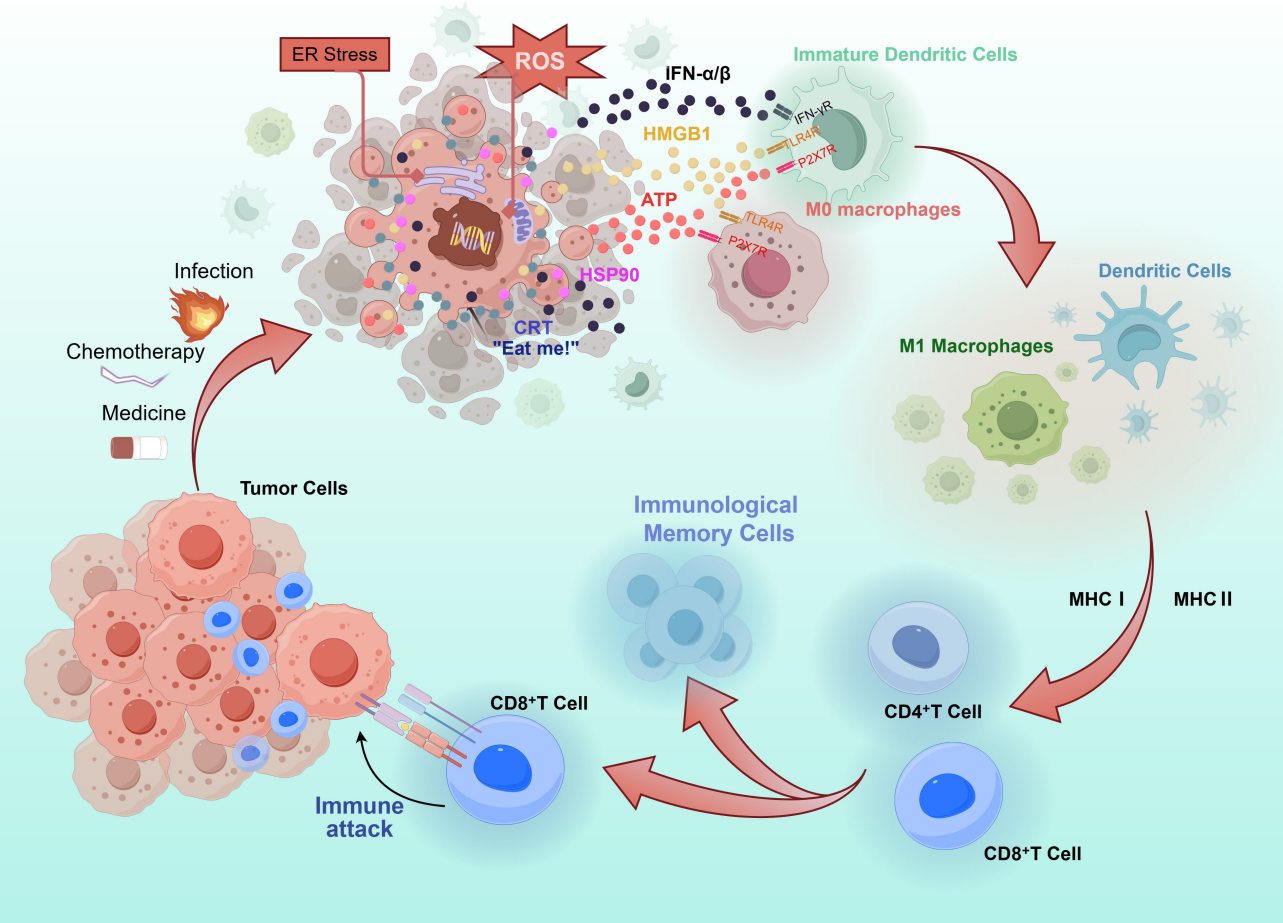
Mechanisms of anti-tumor immune activation triggered by immunogenic cell death. Inducers of immunogenic cell death cause stressed tumor cells to release DAMPs, including surface-exposed CRT, secreted ATP, and released HMGB1 and type I interferons. These signals promote dendritic cell maturation and antigen presentation, and modulate tumor-associated macrophages toward a pro-inflammatory phenotype. Mature dendritic cells migrate to lymph nodes to prime CD4^+^ and CD8^+^T cells, while activated macrophages contribute to tumor microenvironment inflammation and direct tumor clearance. Together, these processes initiate an adaptive immune response leading to tumor-specific T cell attack and eradication of residual tumor cells.

ICD is a form of regulatory cell death. One sign of ICD is the release of molecules released by dead or stressed cells, which can be used as dangerous signals for the immune system. The DAMPs released during ICD include but are not limited to CRT, ATP, HMGB1, IFN-α/β and HSP90/70 (7). The release of DAMPs during ICD significantly enhances antitumor immunity through multiple pathways. These molecules facilitate the uptake, processing, and presentation of tumor antigens by DCs, ultimately leading to the activation of native T cells (**Figure 2**).

### 2.1 Calreticulin

ICD is usually triggered and associated with the ER stress response when activated. In the early stages of ICD, CRT, an endoplasmic reticular companion protein, is crucial for calcium homeostasis and protein folding, and is translocated to the cell surface. PERK, activated by ER stress, promotes erp57-assisted CRT surface exposure, indicating the intake of antigens by DCs, which finally triggers T cell-mediated adaptive immunity (8). This surface-exposed CRT is a powerful “eat me” signal. It is recognized by receptors on antigen-presenting cells (APCs), such as DC or macrophages, which promote the effective engulfing of dying tumor cells and their related antigens. After phagocytosis, the ingested antigens are treated with APC. Antigens of tumor origin are usually cross-presented to CD8+ CTLs through MHC I-type molecules, triggering the adaptive immune response necessary for antitumor immunity (9). Notably, NK cells identify exogenous CRT through NKp46 receptors, which promotes NK cell degranulation and cytokine release, thus helping to remove dead cells (10).

CRT expression is associated with cancer prognosis Surface exposure of cancer cells to CRT is usually associated with favorable results, and high levels of soluble CRT may indicate drug resistance. Specific mutations can completely alter the function of CRT (11). For example, mutations in the C-terminal KDEL ER retention element that destroys CRT, such as CALR del52 and ins5 in myeloproliferative tumors or truncation in solid tumors such as E405*, lead to extracellular secretion abnormalities mediated by the Golgi complex and increase the level of soluble CRT (12). Secreted mutant CRT proteins can function as localized immunological decoys. By occupying receptors on antigen-presenting cells (e.g., CD91), this process hinders the phagocytic capacity of DCs. Consequently, the activation of CD8+ T cells is suppressed, leading to a reduction in the therapeutic effectiveness of both chemotherapy and ICIs.

### 2.2 Adenosine triphosphate

As the primary energy currency within cells, ATP exerts strong immunomodulatory effects during immune cell differentiation. These effects are triggered when ATP molecules are released into the extracellular space via active or passive secretion. In ICD, the release of ATP mainly occurs through the activation of the caspase-dependent Pannexin-1 (Panx1) channel, triggered by ER stress and ROS accumulation, allowing early active secretion when the cytoplasmic membrane remains intact. This is a supplement to passive leakage through membrane rupture during secondary necrosis or through mitochondrial permeable transition pores (mPTP). Extracellular ATP acts on P2 receptors expressed on innate immune cells such as DCs, macrophages and monocytes, and attracts them to the site of cell death as a “find me” signal (13). In addition, ATP activates P2X7 receptors (P2RX7) on DCs. This triggers the formation of NLRP3/ASC/caspase-1 inflammatory bodies, which lead to the secretion of interleukin-1β(IL-1β). IL-1βis crucial for polarization of CD8 + T cells that produce IFN-γ (14). This immunomodulatory effect of ATP not only helps to initiate effective tumor antigen presentation but is also crucial to the synergistic interaction between DC and T cells.

Although ATP plays a key “find me” signaling role in ICD, its metabolite adenosine (ADO) shows strong immunosuppressive properties in the tumor microenvironment (TME) (15). ATP is converted into ADO through the exonucleotide enzymes, CD39 and CD73, which is amplified by HIF-1α under hypoxic conditions (16). ADO mainly binds to P1 purine energy receptors (A1R, A2AR, A2BR, and A3R) (16, 17), inhibits the cytotoxicity of natural killer cells, damages the maturation/antigen presentation of dendritic cells, inhibits the activation of CTLs, and promotes immunosuppressive regulatory T cells (Tregs) and M2 macrophage phenotype, thus driving tumorinogenic activity and exerting a wide range of immunosuppressive effects (15, 18). Accumulation of ADO plays a critical role in driving resistance to ICIs, including agents such as anti-PD-1/PD-L1. This key relationship establishes ADO as a significant and novel regulator of immune checkpoint pathways (19). Therefore, therapeutic strategies targeting the adenosine pathway are being developed, including 1) inhibition of ADO generation (CD39/CD73 blocking), 2) antagonization of ADO receptors, and 3) enhancement of ADO degradation. Considering its relationship with ICI resistance, the combination of ADO and checkpoint pathways (such as the PD-1/PD-L1 pathway) is expected to overcome drug resistance and enhance ICD. Clinical trials such as NCT0345451 have evaluated the A2AR antagonist, ciforadenant, in combination with pembrolizumab.

### 2.3 High mobility group box 1

HMGB1 is a non-histoprotein chromatin-binding protein that is mainly located in the nucleus under physiological conditions, stabilizes DNA structure, and regulates transcription. However, during ICD, HMGB1 is passively released into the extracellular space after the nuclear membrane disintegrates and acts as a key immunostimulatory molecule. Unlike the active secretion of ATP through the Pannexin-1 channel in the early stages of cell death, the release of HMGB1 occurs passively at the end of cell death, after nuclear membrane disintegration. Extracellular HMGB1 participates in PRRs on DCs and macrophages, especially toll-like receptor 4 (TLR4) (13). By combining with TLR4, HMGB1 drives DC maturity and enhances the immunostimulatory ability of DC antigen presentation. In addition, HMGB1 promotes a cross-presentation mechanism, enabling DCs to effectively present tumor antigens to cytotoxic T cells and trigger tumor-specific immune responses. Furthermore, HMGB1 can interact with the receptor for advanced glycation end products (RAGE), thereby promoting the phagocytic clearance of cancer cells by macrophages. The extracellular presence of HMGB1, which often coincides with the amplification of inflammatory signaling, is vital for sustaining the activation of antitumor immunity (20).

Recently, the immunomodulatory function of HMGB1 has been found to be far beyond its initial identification range. In addition to being a classic DAMPs, HMGB1 can also recruit and activate neutrophils to promote the formation of neutrophil extracellular traps (NETs). Under certain circumstances, NETs can enhance antitumor immune responses or promote tumor metastasis (21). It is worth noting that Wang et al. (2022) directly showed that NETs promote the epithelial-stromatic transformation (EMT) and metastasis of non-small cell lung cancer (NSCLC) by downregulating the long-chain non-coding RNA MIR503HG and activating the NF-κB/NLRP3 inflammatory corple pathway, thereby providing new insights into the mechanism of HMGB1-NETs axis in tumor metastasis (22). However, the function of HMGB1 in NETosis is complex. For example, Kunal R More et al. (2025) found that the site-specific mutation of the hmgb1-derived peptide (mB Box-97) can effectively inhibit the activity of protein kinase C (PKC), thus blocking the activation of NADPH oxidase (NOX) and the production of ROS and NETosis. This indicates that targeting the HMGB1-PKC-NOX signaling axis may provide a new therapeutic strategy for suppressing excessive NETosis (23).

In addition, research shows that HMGB1 can be released through non-classical autophagy pathways in a variety of tumors, including gliomas, and regulates the polarization of tumor-associated macrophages (TAM). For example, in glioma cells treated with timozolamide (TMZ), HMGB1 promotes the polarization of TAM to the M1 phenotype through the secretion mechanism of autophagy dependence, thus enhancing the immune response in the tumor and increasing chemotherapy sensitivity (24). In contrast, lactic acid perceived by the GPR65 receptor on TAMs induces the release of HMGB1 through the cAMP/PKA/CREB pathway, accelerating the malignant progression of gliomas (25). Together, these studies show that the release mechanism of HMGB1 and its immune effect depend, to a large extent, on the microenvironment, and that it plays an anti-tumor or tumor-promoting role according to the cell composition, receptor expression, and signaling environment in the TME.

The presence of tertiary lymphoid structures (TLS) in tumors, which are linked to improved immunotherapy outcomes, has also been closely correlated with HMGB1 in numerous cancers (26). From a clinical translation perspective, serum HMGB1 levels are being explored as ICD-induced biomarkers to predict the response to radiotherapy, chemotherapy, or combined immunotherapy, revealing special hope for patients receiving oxaliplatin or radiotherapy.

### 2.4 Type I interferons

ICD uses type I interferon (IFN-α/β) as the key effect molecule, which links tumor cell death with anti-tumor immunity through multiple mechanisms. During ICD, dying tumor cells release mtDNA and activate type I IFN production through the cGAS-STING pathway. Simultaneously, the recognition of dsRNA by TLR3 further amplified this reaction (27). Type I IFNs bind to the IFNAR to activate the JAK-STAT signaling pathway. This cascade promotes the maturation of dendritic cells, enhances the cross-presentation of antigens, enhances the cytotoxic function of NK cells, and inhibits the immunosuppressive function of regulatory T cells, thus establishing strong antitumor immunity (28). In addition, the IFNAR effect of type I IFNs on cancer cells triggers auto secretory and parasecretory signaling pathways. These pathways eventually lead to the transcriptional induction and secretion of interferon-stimulating genes (ISGs), such as T cells, which accumulate the chemokine, CXCL10. This mechanism supports the chemotherapy resistance observed in TLR3- or Ifnar1-deficient tumors (29). However, it is worth noting that type I IFN shows a double effect: acute and strong signals significantly enhance the immunogenicity of ICD, while chronic and low-level signals may promote tumor immune escape (30).

### 2.5 Heat shock proteins

As crucial DAMPs, HSPs released during ICD are pivotal in the regulation of antitumor immunity. When ICD occurs in tumor cells, ER stress can induce upregulation of HSP70 and HSP90 expression, significantly enhancing their immunogenicity (30).

These HSPs can form complexes with tumor-specific antigens and activate the immune system through multiple pathways. On the one hand, these thermoprotein antigen complexes can be identified and internalized by pattern recognition receptors (including CD91, LOX-1 and TLR2/4) on the surface of APC. Subsequently, the antigenic peptide is degraded by the proteasome, transported to the ER through the Tap-dependent pathway, and finally presented as MHC I-type molecules to activate CTLs. On the other hand, the maturation of dendritic cells is facilitated by these complexes via the TLR/NF-κB signaling cascade. This activation leads to an increased expression of co-stimulatory molecules—including CD80, CD86, and CD40—and promotes the production of pro-inflammatory cytokines, like IL-12 and TNF-α. The cytotoxic function of NK cells is augmented by HSP70 through its interaction with the surface receptor, NKG2D (31).

In contrast, HSPs, as key DAMPs, have complex double regulatory effects and are often described as a “double-edged sword”. In cells, members such as HSP70 and HSP90 play a cellular protective role through their partner activity, promote appropriate protein folding and inhibit stress-induced cell apoptosis, thus potentially weakening the antitumor efficacy of chemotherapy and radiotherapy and even promoting tumor cell survival and drug resistance (32). Research has shown that electromagnetic modulation of the TME through an amplitude radio frequency field and other technologies can enhance membrane expression and extracellular release of HSP70. This process works synergistically with other DAMPs, including CRT and HMGB1, to promote DC maturation and antigen presentation, and finally turns the immune response to antitumor phenotype. Therefore, an in-depth understanding of the spatiotemporal expression and functional transformation of HSPs in the process of ICD and the development of strategies to enhance their immune stimulation potential while inhibiting their protective function are crucial to optimize ICD-based cancer immunotherapy.

### 2.6 Spatiotemporal programming of DAMPs

**Figure 3:**
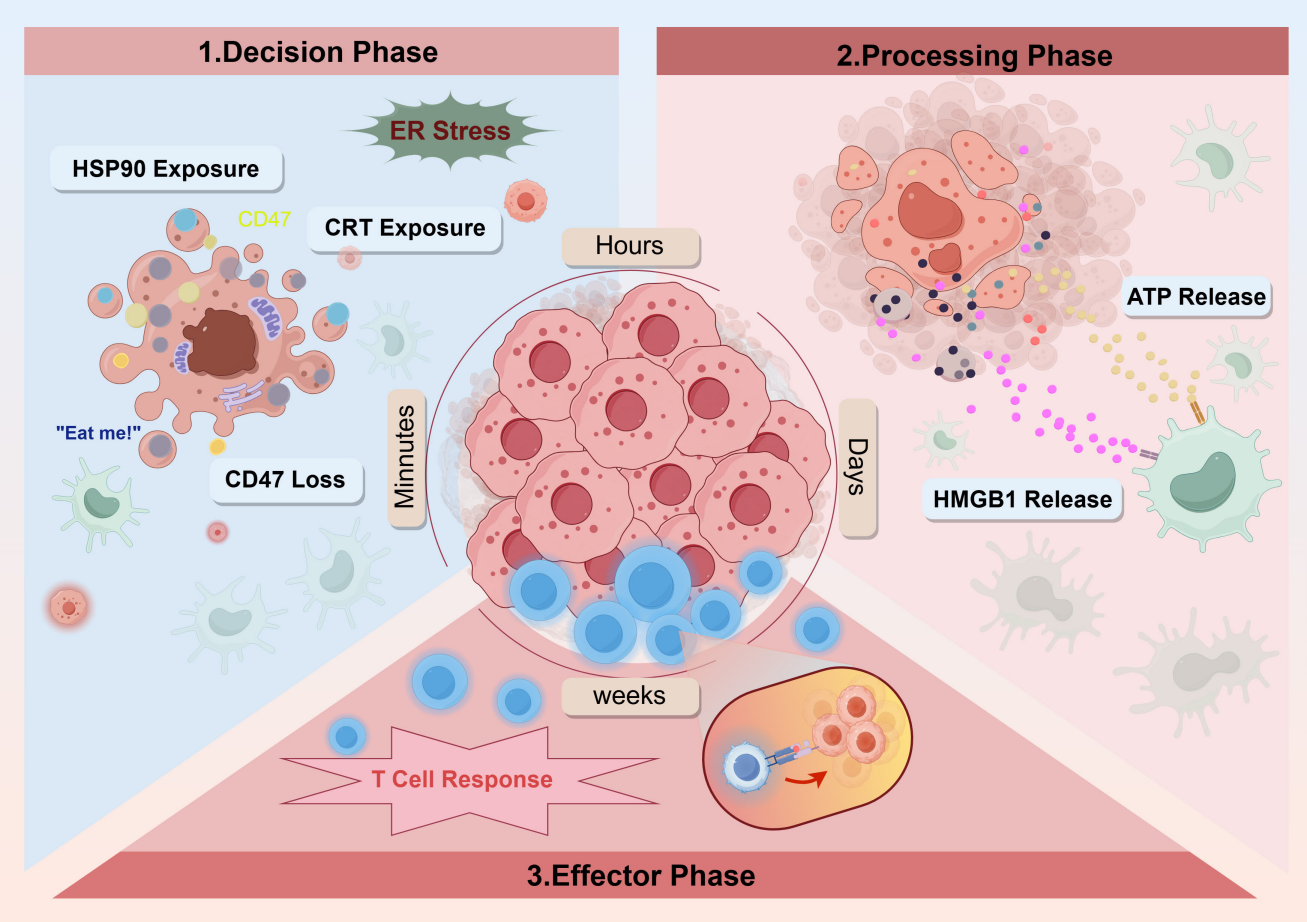
The spatiotemporal programming of DAMPs in ICD. Three-phase space-time cascading reaction of the key DAMPs. The effectiveness of anti-tumor immunity is controlled by three sequential and interdependent stages.

The essence of ICD lies in a highly orderly space-time cascade. Its immune activation effect depends not only on the release of DAMPs, but also on its strict release time. The effectiveness of antitumor immunity is controlled by three stages of space-time procedures: decision-making (signal perception), processing (immune activation), and effect (cytotoxic response) (33) (**Figure 3**).

In the decision stage, CRT is transferred to the cell surface within a few hours of cell death. By binding to phagocyte receptors, membrane translocation of CRT acts as a phagocytic signal for dying cells, enabling DCs to internalize and present tumor-derived antigens. The key is that this phagocytic uptake, which depends on CRT, is enhanced by down-regulating the CD47-a “don’t eat me” checkpoint at the same time; thus, CD47-a protects living cells. This signal modulation begins within 30 min, showing kinetic synchronization with CRT membrane translocation and effective cross-presentation of tumor antigens. Subsequently, HSPs appeared on the surface of dying cells, acting as an adjuvant for tumor antigens and adhesive to DCs. For example, in human myeloma cells, exposure to HSP90AA1 occurs 3–6 hours after apoptotic stimulation (34).

After the DCs successfully engulfs the tumor antigen, the key processing stage begins. At this stage, simple antigen intake is not sufficient to trigger an effective immune response because there is a risk of antigen degradation in lysosomes (35). To start the immune “engine,” two key synergistic signals are needed. As a key “ignition signal,”HMGB1 is released about 18 hours after cell death, binds to the TLR4 receptor on DCs, emits a “danger alarm, “ and triggers downstream immunogenic signals. This signal effectively inhibits antigen degradation in lysosomes, optimizes antigen processing and delivery, and promotes T lymphocyte activation. At the same time, the ATP released during cell stress acts as a powerful “fuel signal.” On one hand, ATP activates NLRP3 inflammatory corpuscles in DCs and promotes the maturity and secretion of pro-inflammatory cytokines such as IL-1β, thus effectively activating CD4+ and CD8+ T lymphocytes. On the other hand, ATP also plays the role of “finding my signal, “recruiting and activating more immune cells, such as mononuclear cells, into the TME. Therefore, the “ignition” of HMGB1 ensures the “quality” of antigen presentation, while the “refueling” of ATP provides the “quantity” of immune activation. In summary, these signals work together to fully activate mature DCs.

During the effect period, mature and fully activated DCs migrate to the lymph nodes, where they effectively present tumor antigens of dead tumor cells to infantile T cells. DCs, which are activated by co-stimulatory and pro-inflammatory signals, orchestrate the differentiation of CD8+ T cells into cytotoxic T lymphocytes and elicit Th1 polarization in CD4+ T cells. These activated T cells are cloned and amplified and then leave the lymph nodes under the guidance of specific chemokines, return to the nest, and infiltrate the tumor tissue. This strong cellular immune response triggered by ICD and centered on IFN-γ is crucial to eliminating tumor cells and establishing effective anti-tumor immunity. More importantly, this process ultimately produces antigen-specific persistent memory T cells, which provide the body with continuous immune surveillance to prevent tumor recurrence and metastasis (35, 36). Therefore, the ICD effect period not only mediates immediate tumor killing, but also lays the foundation for lasting antitumor immunity.

Contrary to their established role, recent evidence has revealed that DAMPs are not solely immunostimulatory; under certain conditions, they can also drive tumor advancement and enable immune evasion (37). For example, in TMEs with high ROS levels, HMGB1 can be oxidized to form disulfide bonds (disulfur HMGB1) and induce beclin-1-dependent protective autophagy through RAGE receptors, significantly reducing the sensitivity of tumor cells to chemotherapy drugs (38). When further oxidized to the sulfonyl form (sulfonyl HMGB1), it completely loses cytokine activity, indicating that the redox state precisely regulates its immune stimulation function (39).On the other hand, extracellular ATP recruits APCs as a “find me” signal at low concentrations; at high concentrations, it may promote the proliferation of regulatory T cells (Tregs) and enhance their immunosuppressive function, or be hydrolyzed into adenosine by CD73, thus forming an immunosuppressive microenvironment (40). Crucially, factors such as hypoxia, acidosis, and oxidative stress in the evolving TME do more than just alter DAMP release kinetics and fine-tune downstream signal transduction and receptor-binding specificity through post-translational modifications. This multilayer regulatory mechanism allows DAMPs to mediate different immune stimulatory effects based on a specific microenvironmental background. Therefore, future ICD-based immunotherapy strategies should establish a multi-dimensional evaluation system that comprehensively considers the molecular morphology, space-time distribution, concentration gradient, and microenvironmental characteristics of DAMPs. This method can accurately predict the direction of immune regulation, avoid potential tumor-promoting risks, and achieve accurate immunotherapy intervention.

## 3 ICD-related cell death pathways

**Figure 4:**
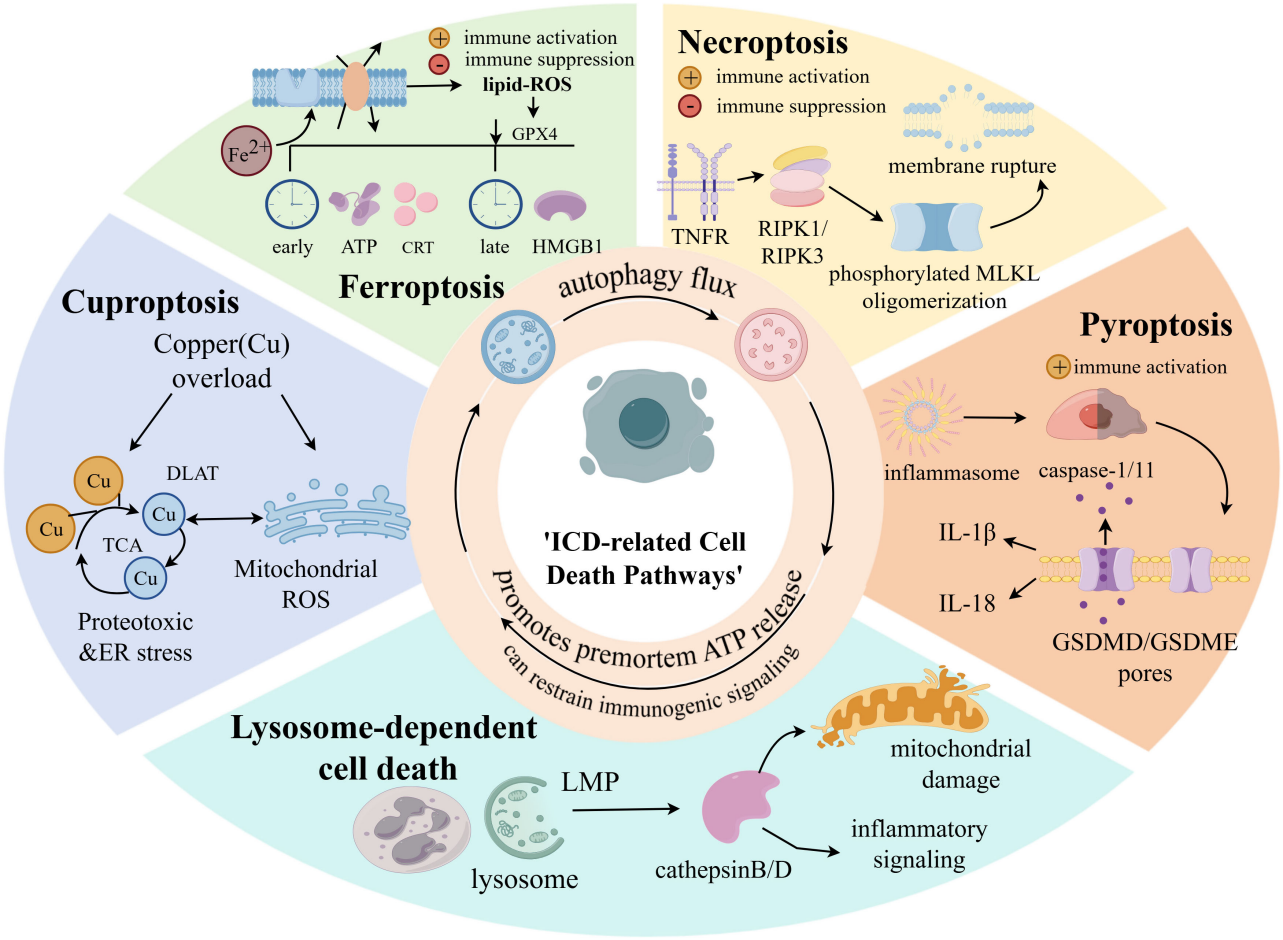
Mechanistic illustration of ICD related cell death pathways. Key regulated cell death modalities that can trigger ICD by promoting the release of DAMPs. Each pathway engages distinct molecular mechanisms: Cuproptosis is initiated by copper overload, which disrupts mitochondrial metabolism by binding lipoylated TCA cycle proteins, leading to proteotoxic stress, ROS generation, and DAMPs emission. Ferroptosis is driven by iron-dependent lipid peroxidation and GPX4 inactivation. Its immunogenic potential involves the context-dependent release of DAMPs such as ATP and HMGB1. Necroptosisis executed via RIPK1/RIPK3-mediated phosphorylation and oligomerization of MLKL, resulting in plasma membrane rupture and passive DAMPs release. Pyroptosis involves caspase-mediated cleavage of gasdermin proteins, forming membrane pores that cause cell lysis and active secretion of cytokines like IL-1β and IL-18.Lysosome-dependent cell death is triggered by LMP, cathepsin release, and subsequent mitochondrial damage, contributing to inflammatory signaling and ICD. The convergence of these pathways on DAMPs release underscores their role in shaping antitumor immunity.

Increasing evidence shows that dying cells actively shape immune responses by releasing dangerous signals that stimulate the innate immune system. However, the relationship between the induction of cell death and the downstream molecular events that determine immunogenicity remains complex. This section discusses the pathways involved in ferroptosis, necroptosis, pyroptosis, cuproptosis and lysosome-dependent cell death, which are usually related to immune activation (**Figure 4**).

### 3.1 Ferroptosis

Driven by iron-dependent lipid peroxidation, ferroptosis is a distinct non-apoptotic mode of regulated cell death. Owing to its unique efficacy in eliminating RAS mutations or chemotherapy-resistant tumor cells, it has received wide attention as a promising tumor treatment strategy (41). Although ferroptosis can induce cell death, its consistency with ICD remains controversial, and its immunomodulatory function shows considerable complexity and environmental dependence.

Several studies have demonstrated that iron subsidence can lead to the release of DAMPs. For example, the release of HMGB1 has been observed in various cancer cell lines treated with ferroptosis inducers. This process depends on the autophagy pathway and has been proven to be significantly inhibited after knocking out ATG5 or ATG7. Notably, HMGB1 released during ferroptosis primarily exerts its immunostimulatory function by binding to the receptor for advanced glycation end products (RAGE/AGER) on APCs, distinguishing it from its toll-like receptor 4 (TLR4)-dependent signaling role in apoptosis. Kinetic analyses indicate that HMGB1 release peaks during late-stage ferroptosis; however, co-culture experiments surprisingly reveal that early stage ferroptotic cells (e.g., after 1 h of RSL3 treatment) were more effective in inducing DC maturation and eliciting protective immune responses, suggesting a critical dependence of immunogenic capacity on the specific phase of cell death (42). Furthermore, HMGB1 participates in regulating ferroptosis, as its deficiency attenuates erastin-induced ROS accumulation and cell death, indicating a dual role for HMGB1 in this process (43). ATP, a key “find-me” signal, is released during the early phase of ferroptosis (1–6 hours) through channels not yet fully elucidated, potentially involving pannexin channels. Extracellular ATP binding to the P2X7 receptor on DCs activates the NLRP3 inflammasome, promoting cytokine production such as IL-1βand IL-18, which subsequently enhance CD8^+^T cell polarization and activation (44). The critical role of ATP is underscored by the finding that blocking P2X7 signaling with oxidized ATP significantly impairs immune protection conferred by early ferroptotic cells in vaccination models. Additionally, surface exposure to CRT has been observed approximately 6 h after ferroptosis induction in prostate cancer models and is associated with anti-tumor immunity *in vivo*. Nevertheless, the molecular mechanisms, precise timing, and relative contribution of CRT exposure to immune recognition during ferroptosis, which are potentially distinct from the rapid ER stress-PERK pathway-mediated translocation characteristic of canonical ICD, require further elucidation. Recent evidence suggests a unique regulatory mechanism for CRT exposure, specifically in ferroptosis. Fan and colleagues made a significant contribution by demonstrating that in oxaliplatin-induced ferroptosis of colon cancer cells, CRT translocation strictly depends on the secretion of high mobility group box 2 (HMGB2) from the nucleus to the cytoplasm, a process regulated by the nuclear export protein, XPO1 (45). They confirmed that inhibiting XPO1 not only blocked the nucleocytoplasmic transport of HMGB2, but also nearly abolished oxaliplatin-triggered CRT surface exposure. Importantly, using a cell-penetrating peptide-targeted HMGB2 (CT-HMGB2), they directly demonstrated that cytosolic delivery of HMGB2 potently induces CRT translocation with an efficacy three orders of magnitude greater than that of oxaliplatin, and that recombinant HMGB2 (but not HMGB1) possesses this function. This revealed a novel “XPO1-HMGB2-CRT” signaling axis for ICD-related exposure, independent of classical ER stress, providing a key molecular explanation for why oxaliplatin, but not cisplatin, induces ICD.

Although the release of these DAMPs provides a theoretical basis for ferroptosis immunogenicity, immunological outcomes are not universally immunostimulatory. However, high-quality research has recently posed significant challenges to its inherent immunogenicity. Wiernicki et al. used a model that allowed the synchronous induction of ferroptosis to divide this process into “initial” (fat ROS accumulation), “intermediate” (ATP release, CRT exposure) and “terminal” (membrane rupture, HMGB1 release) stages, and systematically evaluated each stage for the influence of C function and immune response (42, 46). Their results showed that in the preventive vaccination model, ferroptotic cells could not induce effective antitumor immune protection at any stage. In addition, coculture with ferroptotic cells inhibited DC maturation, antigen cross-presentation, and T cell proliferation. Transcriptomic analysis further showed that the DCs of phagocytic ferroptotic cells showed a significant downregulation of genes (such as *RelB*,*Stat4*,and *Ccr7*) related to T cell activation and the inflammatory response; moreover, the lipid metabolism pathway was abnormally active, resulting in the accumulation of lipid droplets, which then led to impaired antigen presentation function.

These findings indicated that ferroptosis was not intrinsically immunogenic. Its immune consequences are regulated by many factors, including the space-time dynamics of DAMP release, the immunosuppressive effect of byproducts, and the microenvironmental background of the interaction between dying cells and APCs. Fan et al. provided a new perspective: specific stimuli (such as oxaliplatin) may “guide” ferroptosis to an immunogenic fate by activating specific molecular pathways (such as xpo1-mediated HMGB2 secretion), and this process is adjustable. Future research is needed to clarify the key molecular switches that determine the immunogenic fate of ferroptosis and explore joint strategies to use its potential as an effective immunotherapy tool.

### 3.2 Necroptosis

Necroptosis is a strictly regulated form of procedural necrosis that does not depend on caspase activity. It is usually activated, as a backup cell death mechanism, when the apoptotic pathway is inhibited by pathogens or drugs. Its molecular execution is mainly mediated by receptor-interaction protein kinases 1 and 3 (RIPK1 and RIPK3), which eventually leads to phosphorylation, oligomerization, and membrane localization of mixed spectral kinase domain-like protein(MLKL), thus destroying the integrity of the cytoplasmic membrane. This leads to the release of DAMPs, such as HMGB1, ATP and HSP70/90, thus effectively activating the innate immune system (47). In recent years, increasing evidence has shown that necroptosis has considerable immunogenic potential, especially in cancer immunotherapy, and has attracted widespread interest. In contrast to apoptosis, necroptosis, which is characterized by membrane rupture, promotes the release of a large number of DAMPs, effectively promotes the maturation and antigen presentation of DCs, activates the CD8 + T cell response, and in some cases induces systemic antitumor immunity (48).

Recently, the use of necroptosisto enhance ICD has become a new strategy in cancer immunotherapy. Using its unique dissolved death mode, necroptosis drives the release of a wide range of DAMPs, significantly enhancing the antitumor immune response. Based on this principle, Kang et al. developed a bionic Necroptosis-induced nano-vaccine platform (αHSP70p-CM-CaP), which jointly delivers tumor membrane proteins, HSP70 functional peptides, and CpG adjuvants to simulate the DAMPs characteristics of necroptotic cells, thus effectively promoting DC maturity and cross-presentation of antigens (49). In the melanoma model, the vaccine successfully induced a multi-epitopic T cell response and NK cell activation and inhibited tumor growth in conjunction with anti-PD-1 treatment. This study experimentally verified the close relationship between necroptosis and ICD in activating antitumor immunity and provided insights into a new ICD-oriented combined immunotherapy strategy.

Recent studies have indicated that tumor cell migration and invasion can be promoted by necroptosis and prolapse. Li et al. showed that TSZ-induced necrotic cell supernatant can significantly promote the migration and invasion of SCC25 and FaDu cells in head and neck squamous cell carcinoma (HNSCC), while apoptosis did not show this effect (50). Further analysis shows that necroptosis specifically promotes the release of certain DAMPs such as IL-1β, which may drive tumor invasion by activating signaling pathways such as NF-κB.

These results highlight the high contextual dependence of necroptosis’ immunological outcomes. Furthermore, they demonstrate that inter-tumor heterogeneity can influence the effects of DAMPs released from this form of cell death. In some TMEs, necroptosis may cause excessive inflammation or even tumor-promoting effects, such as the accumulation of chronic inflammatory or immunosuppressive cells mediated by HMGB1 (51).

In addition, tumor cells can escape necroptosis and sagging through a variety of mechanisms such as upregulation of the negative regulatory factor of RIPK1 or RIPK3 mutants with missing expression functions, which further increases the complexity of therapeutic targeting of this pathway (52). These observations show that the role of necroptosis in ICD is far from uniform; its net effect depends on a variety of factors, including microenvironmental background, composition of DAMPs, and release kinetics.

In short, the inclusion of necroptosis into the conceptual framework of ICD not only expands our understanding of the immunogenicity of programmed cell death (PCD), but also provides a new way to overcome cell apoptosis resistance. Future research should focus on the development of space-specific necroptosis induction strategies, such as the delivery of RIPK3 agonists or engineering conditions, to activate the genetic circuit using tumor microenvironmental response nanomaterials. At the same time, the integration of single-cell multi-hometics, spatial transcriptomics, and real-time imaging technology is crucial for systematically interpreting the dynamic behavior of necroptosis in the tumor immune microenvironment (TIME) and its series of cell deaths with other forms, thus providing an important scientific basis for accurate immunotherapy applications.

### 3.3 Pyroptosis

Proptosis’s a form of programmed necrotic cell death that is mainly mediated by gasdermin family proteins, especially GSDMD and GSDME, which are activated through the inflammatory pathway of the inflammatory body (caspase-1-dependent) or non-standard pathway (caspase-4/5/11 dependent) (53).Activated cysteine lyase lys GSDMD, resulting in the oligomerization of its N-terminal domain, which then forms pores in the cytoplasmic membrane, eventually leading to cell osmosis and lysis. A key immunogenic feature of pyroptosis is the active release of pre-stored inflammatory cytokines and DAMPs (54). These signals effectively activate DCs and enhance antigen presentation and T cell activation, thus transforming the tumor cell death process into a catalyst to initiate adaptive immune responses. Therefore, pyroptosis is an important form of ICD (3, 55). In addition to its role in ICD, pyroptosis can also be used as a physiological regulatory factor for cell proliferation, stress response, and *in vivo* balance and has a tumor inhibition mechanism. Therefore, induction of pyroptosis has become a promising strategy for antitumor immunotherapy.

ICD caused by pyroptosis works through a multilayer framework. On the molecular scale, the formation of GSDMD or GSDME pores not only causes the leakage of cell components, but also drives potassium discharge, which then activates the NLRP3 inflammatory body and aggravates the inflammatory cascade reaction (56). At the cellular level, extracellular ATP acts as a “find me” signal, attracting APCs to dying cells, and HMGB1 combines with TLR4 on dendritic cells to improve antigen-presentation and co-stimulating molecular expression (57). From the perspective of immunomodulation, thermophages secrete IL-1βand IL-18, which strengthens the function of Th1 differentiation and cytotoxic T lymphocytes, and also improves the expression of MHC I on tumor cells, effectively improving immune alertness (58). In summary, these layered interactions enhance the immunogenic characteristics of dying cells, similar to those of endogenous vaccines.

Interestingly, pyroptosis is not controlled by the typical cysteine pathway. A recent study by Fontana et al. showed that the small-molecule agonist, DMB, directly binds to Cys191 of GSDMD, triggering conformational changes and transmembrane pore assembly, thus bypassing caspase activation of pyroptosis (59).In several mouse tumor models, intermittent low-dose DMB, independent of host immune cells, triggered tumor cell death, resulting in obvious tumor inhibition and strong ICD. Notably, tumor cells exposed to DMB can be used as vaccines to establish a lasting immune memory. When paired with anti-PD-1 treatment, this strategy transforms immune “cold” tumors into “hot” tumors, emphasizing the prospect of its combination.

In addition to GSDMD, GSDME also plays a key role in some tumors. In tumor cells with high GSDME levels, conventional chemotherapy or caspase-3 activation can reduce GSDME and convert cell apoptosis into pyroptosis. Considering the inflammatory characteristics of pyroptosis, this transformation significantly improves immunogenicity (60),thus providing a new theoretical basis for the reuse of established chemotherapies to trigger ICD.

Thus, the role of pyroptosis in cancer remains unclear. Although acute induction can enhance anti-tumor immunity, continuous death may establish a chronic inflammatory environment conducive to tumor growth (61, 62). Therefore, the treatment method must appropriately manage the time and degree of death induction.

In the future, research should give priority to the induction of pyroptosisin space-controlled spaces, such as using nanocarriers that respond to the TME to deliver gasdermin agonists or engineering pre-drugs activated by tumor enrichment proteases. Simultaneously, biomarker-driven patient stratification based on gasdermin expression patterns is crucial for personalized pyroptosis-based interventions. In addition, exploring how pyroptosis induction works together with other immunotherapies (including chimeric antigen receptor T cells and oncolytic viruses) will help overcome immune resistance in solid tumors.

### 3.4 Lysosome-dependent cell death

The immunogenic properties of lysosome-dependent cell death (LDCD) have recently attracted increasing interest. This regulated form of cell death is triggered by lysosomal membrane permeabilization (LMP) (63). After the occurrence of LMP, hydrolytic enzymes (including histoproteinases B and D) leak into the cytoplasm and trigger cell death through a variety of routes, including mitochondrial apoptosis, inflammatory activation, and non-caspase-dependent signal conduction (64, 65). It is worth noting that LDCD does not represent an independent PCD pathway but a terminal event downstream of LMP. The final phenotype (apoptosis, necrosis, or hybridization) is determined by the degree of LMP, cell type, or specific microenvironmental conditions.

Reportedly, certain interventions such as lysosome inhibitors, photodynamic therapy, and nanomaterials can cause immunogenic LDCD (66). For example, Bhardwaj et al. reported that DC661, a dimer chloroquine analog of palmityl protein thioesterase 1 (PPT1), triggers LMP through lysosomal lipid peroxidation (LLP). This leads to tumor cell death and is accompanied by surface exposure to CRT, which promotes the presentation of dendritic cell-mediated antigens and enhances T cell toxicity (67). A key finding was that oxidative stress regulates LDCD immunogenicity because the mechanism of cell death is dependent on ROS accumulation and can be negated by NAC treatment. Further research revealed that the lysosomal cysteine transporter, MFSD12, is crucial for the protection of nac-mediated activity. Deletion of MFSD12 eliminates the ability of NAC to inhibit LLP and LMP, emphasizing the importance of lysosomal redox equilibrium in regulating LDCD (67, 68). The immunogenic effects of LDCD depend on the coordinated release of DAMPs. In addition to CRT exposure, this study recorded active ATP secretion and passive HMGB1 release during LDCD (69).

Nevertheless, the immunological consequences of LDCD are highly environment-dependent. Moderate LMP allows continuous DAMPs release and immune activation, whereas extensive lysosomal rupture may cause sudden leakage of contents, potentially inducing local immunosuppression or tissue damage. Therefore, fine-tuning the size and time of LMP is crucial for enhancing the immunogenic potential of LDCD (70).

In summary, LDCD is an inducible form of ICD and its therapeutic applicability depends on precise LMP regulation. Future studies should aim to clarify the molecular switches that control DAMPs release and immune activation threshold in LDCD and explore the opportunities for synergies with ICIs therapy. Collectively, this knowledge provides a foundation for innovative approaches to multi-agent cancer treatment.

### 3.5 Cuproptosis

As a distinct form of regulated cell death driven by intracellular copper overload, cuproptosis exhibits pronounced immunogenic potential through unique metabolic perturbations and stress-response pathways (71). The fundamental basis of its capacity to induce ICD resides in the tight coupling between mitochondrial dysfunction and ER stress. In contrast to canonical apoptotic pathways, cuproptosis is initiated by the direct interaction of copper ions with lipoylated proteins of the TCA cycle, most notably dihydrolipoamide S-acetyltransferase(DLAT) (72). This interaction induces aberrant oligomerization of mitochondrial proteins and destabilization of iron–sulfur cluster–containing proteins, culminating in severe proteotoxic stress. Such mitochondrial derangements not only compromise cellular bioenergetics but also provoke excessive accumulation of ROS. Transcriptomic profiling and mechanistic investigations consistently demonstrate that this ROS surge functions as a critical upstream signal for the activation of ER stress pathways (71). ROS-driven ER stress subsequently facilitates the active release and surface exposure of DAMPs, including the translocation of CRT to the plasma membrane, extracellular release of HMGB1and ATP, and upregulation of HSPs.

Beyond initiating CD, cuproptosis exerts a self-reinforcing immunomodulatory effect within the TME. On the one hand, copper-based nanotherapeutic platforms can exacerbate oxidative stress by impairing ROS-detoxifying systems, either through inhibition of the NF-κB signaling axis or by direct depletion of intracellular glutathione, thereby amplifying ICD-associated signaling (72, 73). On the other hand, cuproptosis-driven metabolic reprogramming not only enhances DC maturation and antigen cross-presentation but also promotes the phenotypic re-education of TAMs from an immunosuppressive M2 state toward a pro-inflammatory M1 phenotype, concomitant with a reduction in Treg infiltration (71). Collectively, these changes facilitate the conversion of an immunologically “cold” TME into an immune-responsive “hot” state, unveiling novel metabolic–immune targets to overcome resistance to ICB.

Building upon this framework, the ICD cascade initiated by cuproptosis extends beyond localized immune activation to synergize effectively with ICIs, thereby mitigating tumor immune escape. Emerging evidence indicates that cuproptosis induced by copper complexes or ionophores can suppressPD-L1expression on tumor cells via metabolic rewiring, fundamentally attenuating inhibitory signaling toward effector T cells (73). Simultaneously, the release of TAAs and pro-inflammatory mediators during cuproptosis enhances CTL infiltration, limits the accumulation of MDSCs, and further reinforces macrophage polarization toward the M1 phenotype.

Through this multilayered remodeling of the immune microenvironment, cuproptosis inducers function not only as direct cytotoxic agents but also as potent *in situ* tumor vaccines. In addition to eradicating primary lesions, the systemic antitumor immunity elicited can recognize and eliminate distant metastatic foci, thereby suppressing tumor relapse. Accordingly, the combination of cuproptosis-inducing strategies with PD-1/PD-L1blockade has demonstrated markedly enhanced therapeutic efficacy in treatment-refractory tumors. This integrative approach establishes a novel therapeutic paradigm centered on the “metal metabolism–immune regulation “axis, offering promising solutions to the persistent challenges of limited response rates and acquired resistance in cancer immunotherapy.

## 4 ICD inducers in cancer treatment

### 4.1 Chemotherapy

Although traditional chemotherapy exhibits significant tumor cell-killing effects, it induces non-ICD and may even trigger immune suppression, resulting in a lack of sustained therapeutic efficacy. However, recent studies have shown that certain chemotherapeutic drugs such as anthracyclines (doxorubicin, epirubicin, idarubicin, and mitoxantrone), platinum-based agents (oxaliplatin), cyclophosphamide, bortezomib, and bleomycin (74, 75) can induce ER stress and ROS accumulation, thereby promoting the release of DAMPs and triggering an anti-tumor immune response. These DAMPs include exposure to CRT on the cell surface, release of extracellular ATP, secretion of HMGB1, and production of type I interferons, which together constitute the key molecular characteristics of ICD. Among them, doxorubicin (DOX, also known as adriamycin) and epirubicin (EPI, also known as epirubicin) are typical ICD inducers that insert into DNA double strands and inhibit topoisomerase II activity, leading to ROS accumulation and ER stress, thereby activating immunogenic signaling pathways. Preclinical studies have confirmed that tumor cells treated with DOX and EPI can promote DC maturation and antigen presentation through a TLR4-dependent mechanism, thereby enhancing CD8+ T cell-mediated tumor-specific immune responses (76, 77). Notably, animal models lacking *TLR2*,*TLR4*,*or TLR9*genes weakened the therapeutic response to anthracyclic drugs, such as amoxicillin and epiamoxycillin, which emphasizes that the antitumor efficacy of these drugs partly depends on ICD (78, 79).

In colorectal cancer, oxaplatin has also been shown to significantly induce ICD. It activates the ATM/ATR-Chk1/2 pathway after DNA damage, and then activates the PERK-eIF2α-ATF4-CHOP signal cascade (80, 81). Therefore, compared to FOLFIRI (using iliticon plus 5-FU), the FOLFOX regimen (combined with oxaliplatin and 5-FU) showed more effective immunostimulation characteristics (82). Although some drugs, including zemuth and cisplatin, usually do not trigger typical ICDs, they can still promote immunogenic molecular release and DC activation when paired with other treatments (such as radiotherapy or immune checkpoint inhibitors). This synergy ultimately enhances the activity of CTLs in tumors.

In conclusion, these insights not only promote our understanding of how chemotherapy regulates the immune response, but also provide a conceptual framework for the design of ICD-centered combined treatments. By strategically selecting ICD inducers or optimizing their use with non-ICD drugs, it is possible to achieve direct tumor cytotoxicity and induce long-lasting antitumor immune memory. This comprehensive strategy may help improve treatment results and patient prognosis.

### 4.2 Targeted therapy

Currently, the clinical use of targeted anticancer therapy is divided into three main categories: anti-angiogenic drugs (e.g., ramucirumab and aflibercept), epidermal growth factor receptor inhibitors (e.g., cetuximab, panitumumab, and regorafenib), and multi-targeted kinase inhibitors (e.g., regorafenib and fruquintinib) (83). Recent studies have shown that these drugs can not only directly inhibit the tumor growth signaling pathway but also activate the antitumor immune response by inducing ICD. Specifically, cetuximab induces apoptosis of tumor cells by blocking the EGFR signaling pathway and its downstream RAS-RAF-MAPK and PI3K-AKT pathways, and releases DAMPs such as HMGB1 and ATP. These molecules bind to TLR4 and the purinergic receptor, P2RX7, on the surface of DCs to promote DC maturation and activate T cell immune responses (84). In addition, the drug can directly kill tumor cells through antibody-dependent cell-mediated cytotoxicity. HMGB1-TLR4 interaction plays a key role in DC antigen presentation, and ATP promotes IL-1βsecretion by activating NLRP3 inflammatory body, thus driving CD8+ T cell polarization (85).

The antitumor activity of the multi-targeted agent, regorafenib, is mediated by a dual mechanism of action. Its direct effects include the inhibition of angiogenesis and killing of tumor cells. Indirectly, it triggers ER stress to enhance CRT expression and facilitates HMGB1 release via ROS accumulation (86). Regorafenib also promotes the transformation of TAM from tumor-promoting M2 to antitumor M1 (87). Recent evidence suggests that increasing mitochondrial outer membrane permeability (MOMP) stimulates the cGAS-STING pathway, thereby upregulating type I IFN synthesis (85, 88). The platinum n-heterocyclic carbon complex (PtII-NHC) induces the release of DAMPs through ROS-mediated ER stress (89), and antibody-drug couplings(ADCs), such as PBD, mediate immune regulation through CRT and HSP70/90 (90). In addition, in a humanized model of CD30+B cell tumors, brentuximab vedotin treatment promoted the amplification and collection of autologous Epstein-Barr virus-reactive CD8+ T cells, thus enhancing the activity of anti-PD-1 treatment (91). These results demonstrate the potential to induce ICD.

### 4.3 Nanotechnology

Owing to their adjustable physicochemical properties and capacity for surface functionalization, nanomedicines have markedly improved the pharmacokinetics of conventional chemotherapeutic drugs. In addition to enhancing drug stability, solubility, and bioavailability, these systems facilitate tumor-targeted accumulation and controlled drug release by leveraging both the enhanced permeability and retention effects and active targeting mechanisms. As a result, they not only strengthen antitumor efficacy, but also substantially lower systemic toxicity and off-target risks (92). In recent years, nano formulations have garnered considerable attention as potent inducers of ICD. The main mechanisms of nanodrug action can be divided into three categories (93, 94).

(1) Nanocarrier-enabled enhancement of intracellular drug accumulation and stress signaling. By promoting intracellular delivery and tumor-localized exposure of ICD-inducing agents, nanocarriers can intensify ER stress and ROS generation, which cooperatively drive the emission of key DAMPs. For instance, Yang et al. developed an adjustable-size nanoliposome system (PCAL@TM) by linking PTX- and corosolic acid (CA)-loaded nanoliposomes to micron-scale oxygen-carrying liposomes (95). This design enables matrix metalloproteinase-9 (MMP-9)-responsive disassembly and targeted release within the TME, thereby markedly potentiating PTX-induced ICD, as evidenced by increased CRT exposure, HMGB1 release, and ATP secretion. Moreover, by alleviating hypoxia and suppressing inflammatory signaling, this platform helps reverse immunosuppression, ultimately improving therapeutic outcomes of combined PTX chemotherapy and anti–PD-1 immunotherapy (95, 96).
(2) Nanomaterials or nano-carriers that directly trigger and amplify immunogenic signaling. Certain nanomaterials can intrinsically participate in regulating and amplifying immunogenic cues—either through their carrier properties or functional fragments—thereby enabling spatiotemporal control over DAMPs exposure and adjuvant-like signaling. A representative example is provided by Wang et al., who employed the electroactive microorganism Shewanella oneidensis MR-1 to biosynthesize Prussian blue nanoparticles (PB NPs) and subsequently engineered a mitochondria-targeted nanoplatform (MiBaMc) (97). Upon light irradiation, this system simultaneously generates photothermal effects and ROS, resulting in robust ICD-associated signals, including CRT exposure and HMGB1 release, alongside DCmaturation and enhanced T-cell immune activation.
(3) Multifunctional nano-platforms enabling synergistic therapies and immune modulation. Nanotechnology also offers “dual-benefit” designs in which ICD induction in tumor cells is coupled with regulation of APCs, such as promoting DC maturation or polarizing macrophages toward pro-inflammatory phenotypes. A broad array of nanocarriers—including liposomes, nanostructured lipid carriers (NLCs), polymeric micelles, biomimetic membrane-coated nanoparticles—as well as inorganic materials such as metal–organic frameworks (MOFs), gold nanorods, and mesoporous silicon, have been widely explored for delivering ICD inducers (98). These platforms can enhance ER stress, oxidative injury, and mitochondrial dysfunction through optimized biodistribution, TME responsiveness, and co-delivery of multiple ICD inducers or immune adjuvants, thereby amplifying DAMPs signaling and facilitating APC activation. MOF nanomaterials can not only be used as carriers of photosensitizers in photodynamic therapy, but also relieve tumor hypoxia and promote ICD through Fenton-like reactions (99, 100). In contrast, bionic nanoparticles improve the intake efficiency of tumor cells through homogeneous targeting (101). Recent reports have emphasized that some nanoplatforms, such as calcium carbonate-based nanoplatforms, can be degraded under acidic TME conditions, releasing metal ions (such as Cu^2+^). These ions promote the production of ROS through chemodynamic therapy and act together with chemotherapy drugs to induce strong ICD (102).

In summary, nanotechnology not only provides an advanced delivery system for ICD inducers, but can also be used as an immune regulation tool to activate anti-tumor immunity. It has broad clinical application prospects and provides a multifunctional platform for designing next-generation combined immunotherapies.

### 4.4 Physical treatment

Recently, physical therapy (including photodynamic therapy, hyperthermia, radiotherapy, and sonodynamic therapy) has attracted increasing attention because of its ability to effectively trigger ICD and limit systemic toxicity. Several methods are promising for this purpose.

Histotripsy is a non-thermal mechanical ablation technology that uses high-intensity focused ultrasound (HIFU) to produce cavitated nuclei in the target tissue, leading to the formation of bubble clouds. When these bubbles oscillate and burst, the generated mechanical forces can destroy the tumor structure. Recent evidence confirms that tissue sectioning not only causes physical destruction, but also potently triggers ICD, thereby eliciting a systemic antitumor immune response (103). In terms of mechanism, cavitation destroys tumor cell membranes and organelles, and promotes the release of DAMPs. These signals enhance the maturation and antigen presentation of DCs and ultimately promote the infiltration and cytotoxic function of CD8 + T cells in tumors (104). Importantly, because tissue sectioning is independent of thermal effects, it avoids the degradation of heat-related antigens, which helps maintain the integrity of antigens and promotes a more effective specific immune response (105). Given its dual ability of physical ablation and immune stimulation, tissue sectioning may synergize with ICIs or cellular immunotherapy, especially when remodeling immune “cold” tumors, which usually manifests as poor T cell infiltration.

Near-infrared photoimmunotherapy (NIR-PIT) is an emerging strategy for selectively eliminating cancer cells while participating in the host immune system against tumors, showing great clinical potential. This method is based on APCs formed by connecting monoclonal antibodies (mAbs) to water-soluble silicon anhydrous dyes (IR700). These couplings specifically bind to cancer surface antigens. Under local irradiation with near-infrared light (690 nm), IR700 undergoes a photochemical reaction, changes the antibody-antigen complex, destroys the cancer cell membrane, and causes rapid cell death. As a modality of ICD, it mediates both local tumor cell clearance and induction of a host immune response that leads to systemic antitumor immunity (106). Through NIR-PIT-induced ICD, immature dendritic cells mature, activating CD8+ T cells, and enhancing the antitumor immune response. These effects can extend beyond the treatment site and induce distant transfers *in vitro*. In addition, NIR-PIT targets immunosuppressive cells in the TME, such as regulatory T cells, to enhance immune activation (107). This technology is suitable for any type of cancer that overexpresses membrane proteins such as EGFR, HER2, and PSMA, and has shown efficacy in HNSCC, glioblastoma, esophageal cancer, lung cancer, malignant pleural mesothelioma, breast cancer, gastric cancer, and colorectal cancer (106). For each cancer type, one or more specific mAb-IR700 conduits were developed. Intravenous injection of these suffixes, combined with targeted light delivery, can be used to treat a variety of tumors with minimal side effects. Notably, targeted EGFR NIR-PIT has been conditionally approved in Japan and is currently undergoing phase III trials, highlighting its potential for widespread use in cancer treatment in the near future (108).

Tumor treatment field (TTFields) is a noninvasive method that uses a low-intensity alternating electric field at a specific frequency (100–300 kHz) and is currently used for glioblastoma and malignant pleural mesothelioma. The study of Ying Yue et al., using a variety of cell lines, animal models, and immunological analyses, showed that cancer cells treated with TT fields released DAMPs (109). These treated cells are also effectively engulfed by DCs, mature, and upregulate MHC II, CD40, and CD80. In summary, these findings support the conclusion that TTFields induceICD, promote DAMPs release, enhance DC maturation and phagocytosis, and jointly activate antitumor immunity. It is worth noting that TTFields combined with anti-PD-1 treatment appears to produce a stronger immune response, partly because it reverses immunosuppression in the TME and improves the infiltration and function of T cells. A phase III clinical trial (LUNAR trial, NCT02973789) is currently evaluating the combined anti-PD-1 treatment with TTFields, highlighting the transformation prospects of this strategy (94).

Cold Atmospheric Plasma (LTP) is composed of ionized gas near room temperature containing ROS and reactive nitrogen species (RONS). Research shows that LTP can cause tumor cell apoptosis and DNA damage while inhibiting cell viability and migration. In addition, LTP mainly stimulates the anti-tumor immune response by inducing ICD, which supports the development of systemic anti-cancer immunity (110). Therefore, LTP represents a novel and promising method for *in situ* tumor immunotherapy.

Radiotherapy (RT) remains the cornerstone of many cancer treatments, mainly by inducing tumor cell death. More than half of patients with cancer receive radiation therapy for cure or relief. Nevertheless, the radiation resistance of some tumor types remains a clinical obstacle. Reportedly, ionizing radiation (IR) can trigger an antitumor immune response through ICD, although the degree of this effect varies according to radiation type, dose, degree of isolation, and tumor characteristics (111). IR produces ROS, which causes ERstress and leads to the release or exposure of DAMPs. These molecules attract APCs, such as dendritic cells and macrophages. After binding to the pattern recognition receptors on these cells, DAMPs promote APC activation and tumor antigen presentation, ultimately promoting T cell-mediated immunity.

### 4.5 Oncolytic virus

Oncolytic viruses (OV) are a class of tumor-selective viral agents obtained through genetic engineering or natural selection. Currently approved oncolytic viruses include ECHO-7, H101, T-VEC (a recombinant HSV-1 virus expressing GM-CSF), and Teserpaturev. Their mechanism of action involves direct oncolytic effects that trigger ICD-related immune responses (112). After oncolytic viruses selectively infect tumor cells through tumor-specific receptors such as CAR or CD46, they can effectively replicate using the inherent immunodeficiency of tumor cells, eventually leading to cell lysis and the release of a large number of tumor-related antigens (TAAs) (113). In addition, viral-related molecular patterns (VAMPs) unique to oncolytic viruses can identify receptors (PRRs) through patterns such as toll-like receptors (TLRs) and RIG-I/MDA5 to activate type I interferon (IFN-α/β) and pro-inflammatory cytokines (such as IL-6, TNF-α), which can significantly improve the immunosuppressive TME and convert cold tumors into hot tumors (114). Studies have shown that oncolytic viruses expressing CD40 ligands, such as adenovirus, measles virus, and coxsackievirus B3, can induce the above ICD characteristics, and their antitumor effect depends on a fully functional adaptive immune system.

Current research focuses on optimizing the ability of oncolytic virus-induced ICD through genetic modification (such as the expression of immunomodulatory molecules) or combined treatment (such as co-medication with STING agonists or chemotherapy drugs). For example, herpes simplex virus-1 combined with Mito anthraquinone can significantly enhance tumor antigen-specific T-cell responses (115, 116). Despite the limitations and challenges of viral delivery efficiency and immunogenicity regulation, oncolytic viruses have become a remarkable method for cancer immunotherapy. These drugs uniquely combine direct tumor-killing activity and effective immune activation, creating a double treatment mechanism that continues to show remarkable prospects. A wide range of therapeutic agents and modalities capable of inducing ICD are summarized in **Table 1**, providing an overview of their mechanisms and key observations (**Table 1**).

**Table 1:** Examples of available ICD inducers.

Class	Agent	Observations	Ref.
Chemotherapy	Anthracyclines	DOX and EPI induce ICD by DNA intercalation and topoisomerase II inhibition, triggering ROS/ER stress and immunogenic signaling. These agents promote TLR4-dependent DC maturation and antigen presentation, enhancing CD8^+^T cell-mediated antitumor immunity.	(76, 77)
	Oxaliplatin	Oxaliplatininduces bona fide ICD, as validated in both vaccination and established tumor models. This ICD response is mechanistically driven by activation of the DNA damage-triggered ATM/ATR–Chk1/2 axis, culminating in the PERK–eIF2α–ATF4–CHOP signaling cascade.	(140)
	5-FU	5-FU primarily induces ICD through ERstress, in addition to its conventional antimetabolic effect, which ismediated by the inhibition of thymidylatesynthase.	(141)
	FOLFIRI regimen (5-FU + Irinotecan)	Experimental studies in CT-26 colon carcinoma and human tumor models demonstrate that both 5-FU and irinotecan induce ICD hallmarks such as CRT exposure and upregulation of MHC-I molecules. Irinotecan additionally promotes HMGB1 release and enhances dendritic cell maturation via the TLR4/MyD88 signaling pathway.	(82)
	FOLFOX regimen(5-FU +oxaliplatin)	the FOLFOX regimen demonstrates a more potent immunostimulatory effect compared to the FOLFIRI regimen (irinotecan + 5-FU).	(82)
	Actinomycin D	This transcriptional inhibitor induces ICD and is applied in managing pediatric malignancies, including Wilms tumor, rhabdomyosarcoma, and Ewing’s sarcoma, as well as metastatic non-seminomatous germ-cell tumors.	(142)
	Bleomycin	As a DNA-damaging compound, it triggers ICD and is a standard component of regimens for testicular cancer and ovarian carcinoma. carcinoma	(143)
	Cyclophosphamide	This alkylating agent is utilized against breast cancer, certain brain tumors, and hematologic neoplasms, and can promote ICD.	(144)
	Lurbinectedin	A transcriptional inhibitor that induces ICD, recently approved for the treatment of metastatic small-cell lung cancer.	(145)
	PT-112	A novel platinum-pyrophosphate conjugate under investigation, noted for its enhanced efficacy compared to cisplatin in activating the ISR and subsequent ICD hallmarks.	(146)
	Teniposide	A topoisomerase II inhibitor that induces ICD, employed particularly in cases of refractory pediatric acute lymphoblastic leukemia.	(147)
	Aclarubicin	As an anthracycline antineoplastic, it triggers immunogenic stress in tumor cells primarily by potently inhibiting DNA transcription rather than causing extensive DNA damage. Its potency in inducing immunogenic cell death (ICD) is at least equivalent to that of other anthracyclines, yet it significantly lacks the dose-limiting, DNA damage-associated cardiotoxicity characteristic of this drug class. This profile of high ICD induction coupled with lower toxicity may underlie its potentially superior efficacy against cancers such as acute myeloid leukemia.	(148)
Targeted therapy	Cetuximab	Cetuximab triggers ICD by blocking EGFR signaling, releasing DAMPs (HMGB1/ATP). These bind DC receptors (TLR4/P2RX7), stimulating maturation, antigen presentation, and NLRP3‐dependent IL‐1βrelease to polarize CD8^+^T cells. ADCC further kills tumor cells	(85)
	Regorafenib	Beyond its direct anti-tumor effects via angiogenesis inhibition and cytotoxicity, regorafenib promotes immunogenic cell death by inducing ER stress-mediated calreticulin exposure and ROS-driven HMGB1 release.	(86)
	LXR	The LXR agonist T0901317 triggers ICD in colon cancer through CRT membrane translocation and HMGB1 release, leading to potent antitumor immunity and vaccination efficacy in mouse models. Genetic inhibition of CRT or HMGB1 abrogates this protective immune response.	(149)
	PtII-NHC	Compound 2c, a novel platinum-based agent, induces immunogenic cell death in HCC through ROS-mediated endoplasmic reticulum stress and subsequent DAMPs emission. It exhibits superior anticancer activity compared to cisplatin and effectively stimulates immune activation *in vivo*.	(89)
	PBD	The GPC2-targeted ADC D3-GPC2-PBD inducesICD in neuroblastoma by triggering key DAMPs such as calreticulin exposure, HSP70/90 translocation, and HMGB1/ATP release. This promotes DCand T-cell activation, enhances macrophage phagocytosis, and reprograms the tumor microenvironment toward a proinflammatory state, ultimately enabling protective antitumor immunity and synergizing with immunomodulators like CD40 agonists or CD47 blockers.	(90)
	Brentuximab vedotin	Brentuximab vedotin and its payload MMAE induceICD in tumor cells by triggering ER stress and microtubule disruption, leading to innate and adaptive immune activation. These effects synergize with PD-1 blockade, supporting the clinical combination of MMAE-based ADCs with immune checkpoint inhibitors.	(91)
	Belantamab Mafodotin	This BCMA-targeting antibody-drug conjugate delivers a cytotoxic payload, inducing ICD in multiple myeloma cells, and is approved for relapsed/refractory disease.	(150)
	Bortezomib	A proteasome inhibitor that promotes ICD, commonly used in the treatment of multiple myeloma.	(151)
	CDK4/6 Inhibitors	These cell cycle inhibitors exert immunostimulatory effects, including robust interferon signaling, and can contribute to ICD in advanced HR+ breast cancer.	(152)
	Crizotinib	A multi-targeted tyrosine kinase inhibitor that promotes ICD through both intended and off-target mechanisms in non-small cell lung cancer.	(153)
	NUAK1 inhibition	Induces ICD by suppressing the NRF2-mediated antioxidant response, leading to the accumulation of ROS and subsequent release of DAMPs.	(154)
	Mevalonate/Cholesterol pathway inhibition(Simvastatin)	Blocks the negative feedback loop activated by ICD-induced ER stress (via XBnulls). Inhibiting cholesterol biosynthesis prevents the suppression of ROS and DAMP release, thereby amplifying the ICD effect and antitumor immunity.	(154)
Nanotechnology	PB NPs	The mitochondria-targeting nanoplatform MiBaMc, synthesized via biological precipitation, induces ICD in tumor cells through light-triggered amplification of photodamage, leading to tumor antigen release and DCmaturation.	(97)
	PCAL@TM	The size-tunable micro-nano system PCAL@TM delivers paclitaxel and oxygen to lung tumors, where MMP-9-triggered release of nano-liposomes enhances drug penetration and promotes ICD.	(95)
	CMCNs@HA (HA-modified Cu-Mn Composite NM)	Integrates CuO_2_&amp; MnO_2_.Remodels TMEby depleting GSH &amp; generating O_2_.Induces cuproptosis &amp; CDTvia released Cu²^+^/Mn²^+^.Activates robust ICD.Potently stimulates systemic immunityvia cGAS-STING pathway, increasing CD8^+^TILs and inhibiting primary tumor &amp; lung mets. Also serves as activatable T_1_MRI CA.	(155)
	MOF-based NMs	Serve as carriers for PSs in PDT. Can also relieve tumor hypoxia and promote ICD via Fenton-like reactions.	(99, 100)
	CaCO_3_-based systems	Degrade in acidic TME to release metal ions (such asCu²^+^), promoting ROS via CDT and synergizing with chemotherapeutics to induce robust ICD.	(156)
	Liposomes, NLCs, Polymeric Micelles, etc.	Broad array of carriers for delivering ICD inducers. Enhance ER stress, oxidative injury, and mitochondrial dysfunction via optimized biodistribution and TME-R co-delivery.	(157)
	Au NRs, MSNs, etc.	Representative inorganic NMs explored for delivering ICD inducers, often combining PTT properties or high DLC with immune modulation.	(158, 159)
	Copper-Based Composites Nanoparticles	PCD@Cu nanoparticles induce synergistic apoptosis and cuproptosis in TNBC cells, triggering ICD through dual drug release and copper accumulation.	(102)
Oncolytic Virus	ECHO-7, H101, T-VEC (a recombinant HSV-1 engineered to express GM-CSF), and Teserpaturev, and so on.	Clinically approved agents include Rotarix, RotaTeq, and Talimogene laherparepvec.OVs infect and destroy cancer cells, releasing PAMPs/DAMPs and neoantigens, which activate the immune system to trigger inflammation and initiate antitumor immunity.	(112, 113)
Physical treatment	Histotripsy	histotripsy not only directly ablates tumors but also may activate ICD-related immune responses both *in vitro* and *in vivo*.PFP-loaded nanodroplets enable controlled bubble cloud cavitation under low-intensity ultrasound, effectively inducing ICD through DAMPs release and immune cell infiltration.	(103)
	NIR-PIT	NIR-PIT induces rapid and selective ICD by targeting cancer-specific antigens, triggering dendritic cell maturation and multi-clonal cytotoxic T cell responses.	(107)
	TTFields	TTFields therapy induces ICD in cancer cells by triggering DAMPs release and CRTexposure, thereby activating DCs and promoting immune cell infiltration.	(109)
	Radiotherapy(irradiation)	Potent ICD inducer that is widely employed in the management of individuals with cancer	(160)
	Extracorporeal photochemotherapy(8-methoxypsoralen)	ICD-inducing photosensitizer commonly employed for the extracorporeal treatment of cutaneous T cell lymphoma	(161)
	Photodynamic Therapy (PDT)	PDT utilizes photosensitizers such as hypericin, redaporfin, and verteporfin to induce ICD. These agents hold orphan drug status or are under clinical development for malignancies including cutaneous T-cell lymphoma and cholangiocarcinoma.	(162)

## 5 ICD-based combination therapies

### 5.1 Combination therapy with ICIs

ICIs have marked revolutionary progress in cancer immunotherapy, providing a continuous clinical response in a series of malignant tumors. However, their effectiveness is often limited by the immunosuppressive nature of the TME, which is characterized by insufficient infiltration of cytotoxic T lymphocytes, T cell exhaustion, and accumulation of immunosuppressive cells (117, 118). Against this background, ICD has attracted attention as a promising method for reprogramming the TME through multiple mechanisms to combat ICI resistance.

Clinically, chemotherapy combined with ICIs has repeatedly achieved better results in cancers, such as non-small cell lung cancer, melanoma, and triple-negative breast cancer. This synergy seems to stem from chemotherapy-induced ICD and ICIs, which enhance antitumor immunity (117). For example, the well-characterized ICD inducer, oxaliplatin, can not only cause DNA damage, but also activate ATM/ATR-Chk1/2 and PERK-eIFα-ATF4-CHOP pathways. These pathways stimulate CRT exposure, HMGB1 release, and ATP secretion, thus participating in the DC-CD8 + T-cell axis (81). According to Pfirschke et al., pretreatment with oxaliplatin greatly increases the infiltration of CD8 + T cells into tumors and cooperates with PD-1 inhibitors to inhibit tumor growth. This result has also been observed in several solid tumor models (119).

Encouraging preclinical data has accelerated the clinical adoption of continuous “ICD activation-ICI enhancement” schemes. Many phase III trials have strengthened the value of combining ICD-induced chemotherapy with ICIs for the treatment of various cancer types. For example, the KEYNOTE-189 trial reported that the combination of pimumab with platinum-based chemotherapy significantly improved progression-free and total survival of patients with non-small cell lung cancer (120). Similarly, the IMpassion130 trial reported a significant increase in the survival rates of atezolizumab and nab-paclitaxel in the treatment of triple-negative breast cancer, especially in PD-L1-positive individuals (121). The KEYNOTE-522 study further showed that the new adjuvant, pimumab, plus chemotherapy could significantly improve the pathological complete remission and event-free survival rate of early triple-negative breast cancer (122).

Therefore, a successful combined treatment outcome is contingent upon key variables such as the chemotherapeutics used, planned regimen, and the heterogeneous nature of the tumor. For example, the KEYNOTE-361 trial failed to reach the main endpoint of advanced urinary tract cutaneous carcinoma, emphasizing that not all chemotherapies can effectively induce ICD or cooperate effectively with ICIs (123). In the future, it will be very important to improve the ICD induction scheme by identifying effective inducers such as oxaliplatin and anthracycline drugs, determining the optimal dose and time, and using biomarker-guided patient selection (for example, through PD-L1 expression, tumor mutation burden, or DAMPs release spectrum) to achieve precise immunooncology.

In summary, the combination of ICD inducers and ICIs is a biologically reasonable and clinically feasible immunotherapeutic strategy. Future research should use ICD-regulated space-time analysis, multi-group datasets, and artificial intelligence-driven tools to tailor this strategy for personalized cancer treatment, with the ultimate goal of improving patient prognosis.

### 5.2 Combination therapy with cyto-therapy

In the treatment strategy of combining cell therapy with ICD, chimeric antigen receptor T-cell (CAR-T) treatment has shown breakthrough progress and significant efficacy in the treatment of recurrent/intractable malignant blood diseases. So far, more than 10 CAR-T products have been approved worldwide, mainly targeting B-cell markers such as CD19 and BCMA, for the treatment of non-Hodgkin’s lymphoma, acute lymphocytic leukemia and multiple myeloma. Its mechanism of action depends on genetically engineered T cells that express specific CAR structures on their surfaces. These cells activate downstream signaling pathways by identifying tumor-related antigens, releasing perforin, granulase B and other effect molecules to directly kill target cells, and secreting various cytokines (such as IFN-γ, IL-2) to recruit and activate endogenous immune cells, and finally establish long-term immune memory to prevent tumor recurrence (124).

Recent studies have shown that ICD inducers have potential applications in the treatment of blood tumors. As blood tumor cells are distributed in the peripheral blood and bone marrow microenvironment, drugs are more likely to reach and induce ICD through the circulatory system, triggering the release of critical DAMPs. These molecules bind to receptors such as CD91, TLR4, and P2RX7 on DCs, thereby promoting DC maturation, antigen cross-presentation, and CD8+T cell polarization. Therefore, the activation and amplification of CAR-T cells are enhanced (125). Additionally, the high specificity of antigens expressed in blood tumors (such as CD19, BCMA, and CD138) enables CAR-T cells to accurately identify and support effective and targeted removal.

However, the effectiveness of CAR-T cells in treating solid tumors is limited by several obstacles. These factors include the heterogeneity of target antigen expression, infiltration restriction caused by the matrix barrier, and T cell failure or function inhibition in the immunosuppressive TME (126). ICD coordinates the release of multiple DAMPs to reshape the TME. This not only enhances the antigen presentation ability of DCs, but also promotes chemochemistry and infiltration of T cells through chemokine signal conduction, which helps to reverse local immunosuppression. For example, HMGB1 enhances the expression of MHC-I and co-stimulated molecules in DCs through TLR4 signaling, thereby improving the antigen recognition efficiency of CAR-T cells. Simultaneously, ATP activates the NLRP3 inflammatory body, inducesIL-1βsecretion, and supports the differentiation of effector T cells.

Emerging preclinical evidence shows that the combination of ICD induction strategies (chemotherapeutic drugs, such as oxaliplatin and amoxicillin, or nanocarrier-based delivery systems) with CAR-T treatment can significantly improve the infiltration and persistence of CAR-T cells in solid tumors. For example, in the hepatocellular carcinoma model, ICD induction leads to a significant increase in the number of local CAR-T cells and IFN-γsecretion, accompanied by a decrease in the proportion of Treg. This indicates that ICD may improve the efficacy of CAR-T cells by altering the composition of immune cells (127). In addition, engineered “armored” CAR-T cells that secrete IL-12 or express dominant negative TGF-β receptors can be further coordinated with ICD signals to fight immunosuppressive TME and establish a positive feedback loop in anti-tumor immunity.

It is worth emphasizing that CAR-T therapy and ICD strategies have a multilevel synergy. On the one hand, ICD-mediated antigen release and DC activation can broaden the antigen recognition spectrum and affinity of CAR-T cells. On the other hand, CAR-T cells directly exert cytotoxicity, further release tumor antigens, and produce the “antigen diffusion” effect, thus expanding the immune response. Additionally, ICD-related DAMPs can activate the cGAS-STING pathway, induce type I interferon production, and enhance cell function and immune surveillance (128). In a preclinical model, the combined use of ICD inducers (such as oxaliplatin) and CAR-T cell treatment not only increased the number of tumor-infiltrating CAR-T cells but also prolonged their survival time in the TME (129). This suggests that ICD may help delay T-cell failure by improving metabolic conditions and mitochondrial function.

In addition to CAR-T cells, the combination of ICD with other car-engineered immune cells also has unique advantages. For example, ICDs show special prospects in Chimeric antigen receptor macrophages (CAR-M)treatment. Macrophages are the most abundant immune cells in the TME and exhibit strong tissue penetration and antigen presentation abilities. CAR-M cells can directly engulf tumor cells and secrete MMPs to decompose the extracellular matrix, thereby enhancing the infiltration of immune cells (130). When used in combination with ICD inducers, the DAMPs released by tumor cells further promote the polarization of CAR-M cells to pro-inflammatory M1 phenotype, enhance their antigen presentation function, and stimulate the secretion of cytokines such as TNF-α, and jointly reshape the immunosuppressive microenvironment. CAR-M constructs bound to the intracellular TLR4 signal domain significantly upregulate co-stimulating molecules, such as CD86 and MHC-II, during ICD stimulation, accelerating tumor regression (130, 131). Currently, the CAR-M drug MT-101, which targets CD5, has entered clinical trials for the treatment of recurrent/intractable T-cell lymphoma (NCT05138458), providing preliminary clinical support for the combined treatment strategy of ICD-CAR-M.

Emerging research points to the particular promise of integrating ChimericAntigenReceptorNaturalKillercelltherapy(CAR-NK cell therapy) with ICD, partly because CAR-NK cells present a diminished risk of complications, such as cytokine release syndrome and graft-versus-host disease, compared to that with similar treatments, such as CAR-T cell therapy. Their cytotoxic activity does not require antigen pre-sensitization and can be enhanced by antibody-dependent cytotoxicity mediated by CD16. In a recent study by Zheng et al., ERp57, an ER protein co-displaced to the cell surface with calciferous proteins during ICD, was identified as an ideal target for CAR-NK cells (132). Researchers have isolated the nanobody G6 with high affinity for both human and mouse ERp57 from the camel VHH bacteriophage display library and constructed CAR-NK92 cells targeting ERp57. They confirmed the expression of ERp57 on the baseline surface of a variety of tumor cell lines, which was further enhanced by membrane translocation after low-dose oxaliplatin treatment. *In vitro*, G6-CAR-NK92 cells effectively killed ERp57-positive tumor cells, and cytotoxicity was significantly enhanced after oxaliplatin-induced ICD. In hematology and solid tumor xenotransplantation models as well as in patient-derived xenotransplantation models, G6-CAR-NK92 cells showed strong antitumor activity, which was further enhanced when used in combination with oxaliplatin. In terms of mechanism, ICD inducers increase the surface exposure of ERp57 on tumor cells, improve the identification and infiltration of CAR-NK cells, and also promote the release of effect molecules such as granulase B, IFN-γ and TNF-α.

This study introduces a new model that combines ICD with cell therapy: using the ER protein specifically transferred to the cell surface during ICD as an “induction target.” This method can accurately identify and eliminate tumor cells, while minimizing off-target toxicity in normal tissues. It is especially suitable for solid tumors with high antigen heterogeneity, because ICD inducer pretreatment can dynamically regulate target expression and enhance the nesting and killing effects of CAR-NK/T/M cells.

DCs are central to the immune response initiated by ICD are DCs, which function as the body’s premier professional APCs. A significant advancement is chimeric antigen receptor dendritic cells (CAR-DCs), which utilizes the CAR structure to target and phagocytose tumor cells. Furthermore, these engineered cells competently process and cross-present tumor antigens, thereby stimulating a powerful cytotoxic T lymphocyte response (131). When used in combination with ICD inducers, they may work together to promote the maturation of CAR-DCs and their migration to lymphatic tissues. A clinical trial evaluating the combined treatment of recurrent/intractable B-cell lymphoma (NCT05585996) with CD19-targeted CAR-T and CAR-DC cells is underway to explore the clinical feasibility of this synergistic strategy.

Future research should focus on identifying other ICD-related induction surface targets, optimizing the CAR signal structure, and determining the optimal time and dose coordination between the ICD-inducer dose and CAR cell infusion. Combined with single-cell sequencing and spatial transcriptomics, a systematic analysis of the kinetics of the immune microenvironment in the combined treatment process helps lay the theoretical foundation for combined immunotherapy of accurate and personalized ICD cells.

### 5.3 Combination therapy with gut microbiota

Gut microbiota metabolites, which serve as crucial mediators of the interaction between gut microbes and the host, play a significant role in regulating ICD and enhancing anticancer therapies. These metabolites primarily include short-chain fatty acids (SCFAs, such as acetate, propionate, and butyrate), tryptophan derivatives (e.g., indole-3-aldehydeand kynurenine), and bile acid derivatives (e.g., deoxycholic and lithocholic acids) (33). Studies have demonstrated that these metabolites significantly enhance ICD effects induced by conventional therapies through epigenetic regulation and immunomodulatory mechanisms. For instance, butyrate promotes the expression of key ER stress molecules, PERK and eIF2α,by inhibiting HDAC activity and increasing oxaliplatin-induced CRT membrane translocation levels by 3-fold (p<0.01) in colorectal cancer. Propionate, via the activation of G protein-coupled receptor 43 (GPR43), enhances radiotherapy-induced ATP release, increasing CD8+ T cell infiltration by 40% in a melanoma model (133).

Based on these properties, the combination of gut microbiota modulation and ICIs has demonstrated significant synergistic effects. Furthermore, gut microbiota metabolites like butyrate can convert “cold tumors” into “hot tumors” through epigenetic regulation of PD-1 expression, thereby expanding the applicable population for ICIs. Bifidobacterium enhances PD-1 inhibitor-induced tumor antigen presentation by activating the TLR4-MyD88 signaling pathway in DCs (134), whereas *Akkermansia muciniphila* significantly improves the response to PD-1 blockade therapy by upregulating the recruitment of CD4+ T cells. It is also noteworthy that specific strains, such as Bifidobacterium longum, activate DCs via the TLR2/4-MyD88 signaling pathway, significantly enhancing 5-fluorouracil-induced ICD effects (133). *Bacteroides fragilis* improves breast cancer treatment efficacy by increasing doxorubicin-induced HMGB1 release 2.5-fold through the TLR4 signaling pathway. These findings provide a molecular basis for microbiome modulation to enhance the effects of conventional ICD therapies.

Furthermore, gut microbiota metabolites exhibit significant temporal and spatial complementarity with oncolytic viruses to jointly induce ICD (135). Metabolites such as SCFAs systematically improve the TIME by regulating dendritic cell maturation and T-cell differentiation. In contrast, oncolytic viruses locally activate the TLR3/RLR signaling pathway via viral pathogen-associated molecular patterns (PAMPs), synergistically enhancing ICD effects. Preclinical studies have demonstrated that in malignant glioma models, gut microbiota preconditioning significantly increases the tumor-targeting efficiency of oncolytic viruses by 2-fold (p<0.05) compared to that in controls. Concurrently, the virus-induced inflammatory response promotes the colonization of beneficial bacteria, such as *Akkermansia muciniphila*, establishing a positive immunomodulatory cycle. Several clinical trials have evaluated the value of this combination therapy. Microbiota-virushybrid systems developed using synthetic biology techniques, such as engineered viruses expressing SCFA-synthesizing enzymes, have the potential to achieve more precise ICD modulation (136). These advances have provided novel strategies for overcoming the resistance to cancer immunotherapy. However, key challenges, including metabolite delivery efficiency, individualized microbiota variations, and safety of combination therapy, must be addressed before clinical translation.

### 5.4 Combination therapy with oncolytic viruses

Preclinical studies have shown that oncolytic viruses (OVs) not only enhance local antitumor immune responses by inducing PCD but also trigger distant effects, providing a theoretical basis for their combination with radiotherapy, chemotherapy, or ICIs. Genetically engineered OVs, such as T-VECs expressing granulocyte-macrophage colony-stimulating factor (GM-CSF) or oncolytic viruses carrying PD-1 antibodies (120), exert their effects via a dual mechanism. On one hand, it enhances the recruitment and activation of DCs. In contrast, blocking immune checkpoint signaling pathways significantly enhances the synergistic immune effects of ICD. Specifically, oncolytic viruses induce ICD by releasing tumor-associated antigens (TAAs) and DAMPs, thereby increasing the infiltration of CD8+ T cells in TME and upregulating the expression of PD-L1, transforming “cold tumors” that were previously unresponsive to ICIs into immune-sensitive “hot tumors.” Clinical trials have demonstrated that this synergy translates into patient benefits. A specific melanoma regimen was sequentially administered using T-VEC, a modified oncolytic herpes simplex virus, followed by pemumab immunotherapy. The objective remission rate was 62%, significantly exceeding that of single-drug treatments (137). These results highlight the potential of combined immunotherapy based on OVs; however, improved dose sequences and treatment regimens are necessary to achieve clinical benefits.

**Figure 5:**
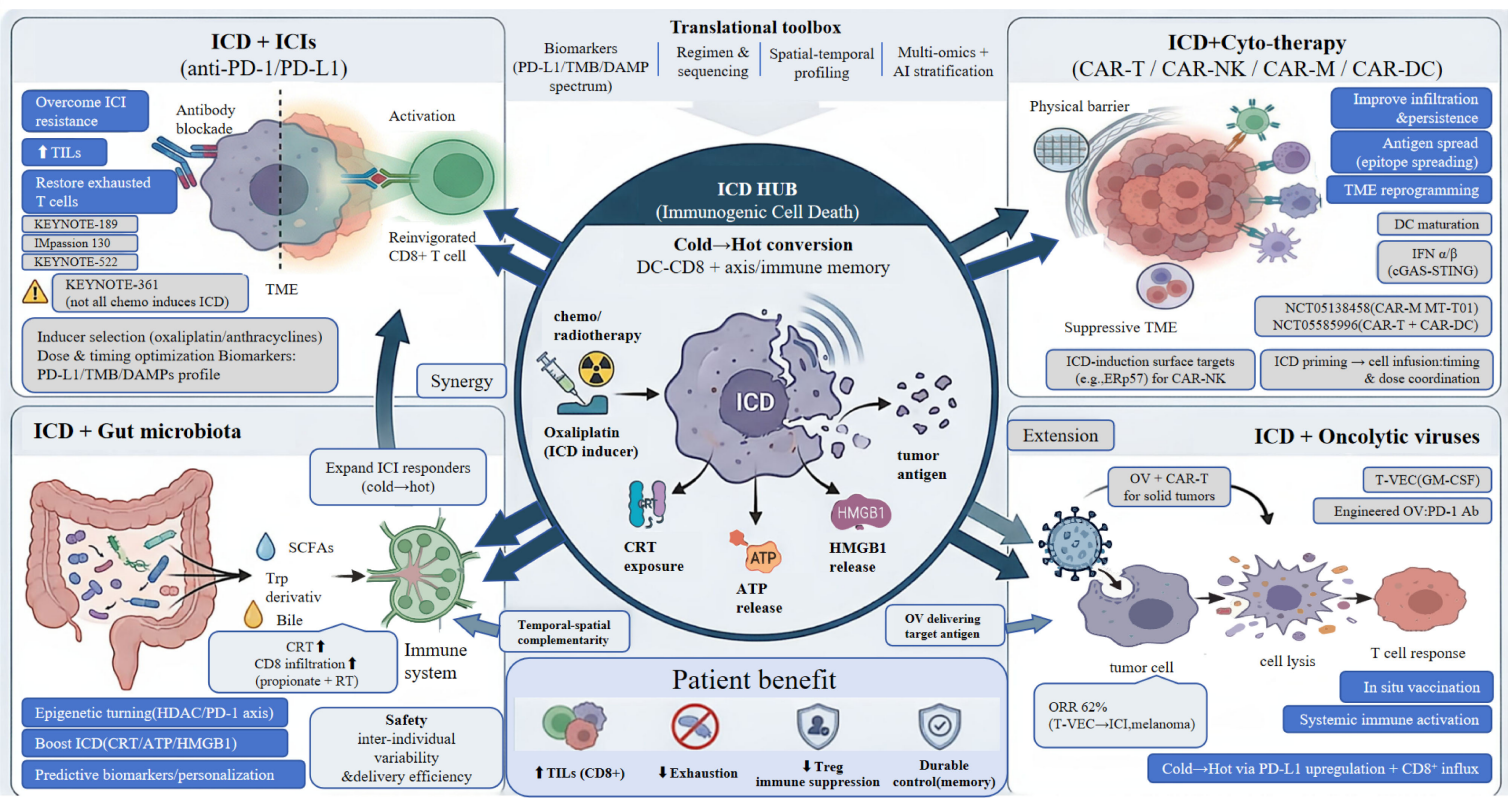
ICD-based combination therapies: A Synergistic Hub for Next-Generation Cancer Immunotherapy. ICD acts as a central mechanism to convert immunologically “cold” tumors into “hot” ones, primarily by triggering the release of DAMPs that activate dendritic cells and promote cytotoxic CD8^+^T-cell responses. This positions ICD as a strategic hub to enhance multiple immunotherapeutic modalities. (1) ICD + Immune Checkpoint Inhibitors: ICD inducers (such as oxaliplatin) overcome ICIs resistance by recruiting TILs, reversing T-cell exhaustion, and transforming “cold” tumors into ICIs-responsive “hot” tumors, as demonstrated in clinical trials (KEYNOTE-189, IMpassion130). (2) ICD + Cell Therapy (CAR-T/NK/M/DC): ICD remodels the immunosuppressive tumor microenvironment, improving the infiltration, persistence, and efficacy of CAR-engineered cells against solid tumors, with several clinical trials underway (e.g., NCT05138458, NCT05585996). (3) ICD + OVs: OVs function as *in situ* vaccines, inducing ICD and systemic immune activation. Engineered OVs (such as T-VEC) synergize with ICD to enhance ICIs responses and are being explored in combination with CAR-T cells. (4) ICD + Gut Microbiota: Microbial metabolites (such as short-chain fatty acids) epigenetically enhance ICD effects, promote anti-tumor immunity, and expand the population of ICIs responders. Translational Integration: Successful implementation relies on biomarker-driven patient stratification (such as PD-L1, TMB, DAMPs profiles), spatiotemporal profiling, and optimized treatment sequencing. Overall Impact: These synergistic strategies aim to improve clinical outcomes by enhancing TILs, reducing immunosuppression, and establishing durable anti-tumor immunity.

Combined treatment with OVs and CAR-T cells is a promising approach for treating solid tumors. By inducing ICD, OVs trigger the release of TAAs and DAMPs, thereby reshaping the immunosuppressive TME and promoting the collection, activation, and amplification of CAR-T cells (125). In addition, OVs can be designed to express specific tumor-targeted antigens, effectively acting as “marker” carriers to improve CAR-T cell recognition and eliminate malignant cells. Reportedly, the synergy of OVs and CAR-T cells can offset the immunosuppressive properties of the TME, thus producing stronger and more continuous antitumor immunity (138). For example, in animal models, the design of an oncolytic adenovirus expressing CD19 resulted in significant tumor suppression and prolonged survival (139). In summary, **Figure 5** provides a comprehensive visual summary of these synergistic strategies, highlighting how ICD integrates with immune checkpoint inhibitors, cell therapies, gut microbiota, and oncolytic viruses to remodel the tumor microenvironment and enhance antitumor immunity (**Figure 5**).

## 6 Conclusions and future perspectives

Although ICD has reshaped tumor immunotherapy, its clinical translation still faces several challenges.First, human evidence and available drugs remain limited. Only a small number of ICD inducers are routinely used in the clinic, and many promising agents are still confined to *in vitro* and animal studies. As a result, robust, direct proof that patient benefit is truly driven by ICD is still scarce. Second, toxicity and suboptimal dosing strategies constrain efficacy. Most established ICD inducers are traditional cytotoxic agents with broad off-tumor toxicity, including damage to immune cells. Conventional “maximum tolerated dose” regimens can induce systemic immunosuppression. This highlights the need for optimized dosing and schedules that preserve or even enhance antitumor immunity rather than suppress it. Third, biomarker and assay systems are underdeveloped. There is still no standardized, clinically validated ICD biomarker panel or scoring system suitable for routine practice. Existing recommendations mainly serve preclinical screening. Classical DAMPs such as calreticulin exposure, ATP release, and HMGB1 release are not specific for ICD, are difficult to measure reproducibly in patients, and their simple presence or expression does not necessarily indicate true extracellular emission or functional immune activation.

Future research should prioritize several key directions. First, personalized ICD induction strategies must be developed through multigroup analyses and artificial intelligence-driven reaction prediction. Using new delivery platforms, such as stimulating reactive nanocarriers and engineered exocrine bodies, enhanced targeting can be achieved. Second, it is important to have an in-depth understanding of the synergistic mechanism between ICD and other immunotherapies (such as immune checkpoint inhibitors, CAR-T cell therapy, and microbiome regulation). This will help optimize the time and sequence of combined medications. In addition, the establishment of a dynamic monitoring system integrating liquid biopsy and functional imaging as well as a standardized biomarker evaluation framework will enable the evaluation of ICD efficacy more accurately and in real time.

It is also worth noting that future research must solve how to balance the immune activation effect of ICD and the risk of excessive inflammation, while overcoming the immunosuppressive TME. With the development of synthetic biology, spatial multigroup, and organ-like models, ICD research is expected to achieve more accurate space-time control. This progress will help bridge the gap between laboratory findings and clinical practice, and ultimately provide curative immunotherapy to a wider group of patients.

## Glossary

ICD: Immunogenic Cell Death
DAMPs: Damage-Associated Molecular Patterns
ADO: Adenosine
APC: Antigen-Presenting Cell
ATP: Adenosine Triphosphate
CAR: Chimeric Antigen Receptor
CRT: Calreticulin
CTL: Cytotoxic T Lymphocyte
DC: Dendritic Cell
EMT: Epithelial-Mesenchymal Transition
PRRs: pattern recognition receptors
ER: Endoplasmic Reticulum
HCC: Hepatocellular Carcinoma
TLS: tertiary lymphatic structure
HMGB1: High Mobility Group Box 1
ICI: Immune Checkpoint Inhibitor
IFN: Interferon
IL: Interleukin
MHC: Major Histocompatibility Complex
NET: Neutrophil Extracellular Trap
NK: Natural Killer Cell
NSCLC: Non-Small Cell Lung Cancer
TAM: tumor-associated macrophages
ISGs: interferon-stimulating genes
Treg: Regulatory T Cell
HMGB2: high mobility group box 2
HNSCC: head and neck squamous cell carcinoma
MHC: major histocompatibility complex
Panx1: Pannexin-1
mPTP: mitochondrial permeable transition pores
P2RX7: P2X7 receptors
LDCD: lysosome-dependent cell death
LMP: lysosomal membrane permeabilization
LLP: lysosome lipid peroxidation
OV: Oncolytic Virus
PD-1: Programmed Cell Death Protein 1
PD-L1: Programmed Death-Ligand 1
PRR: Pattern Recognition Receptor
MOFs: metal-organic frameworks
RAGE: Receptor for Advanced Glycation End-products
ROS: Reactive Oxygen Species
SCFA: Short-Chain Fatty Acid
TAA: Tumor-Associated Antigen
TLS: Tertiary Lymphoid Structure
TME: Tumor Microenvironment
TLR: Toll-Like Receptor
TNF: Tumor Necrosis Factor
PAMPs: pathogen-associated molecular patterns
VAMPs: Virus-Associated Molecular Patterns
Au NRs: Gold Nanorods
CA: Carbonic Anhydrase/Cinnamic Acid (context-dependent)
CDT: Chemodynamic Therapy
CIT: Chemoimmunotherapy
CuO₂/MnO₂: Copper Dioxide/Manganese Dioxide
DLC: Drug Loading Capacity
DOX: Doxorubicin
ex-srNPs: Exogenous Stimuli-Responsive Nanoparticles
GSH: Glutathione
HA: Hyaluronic Acid
IDO1i: Indoleamine 2,3-Dioxygenase 1 Inhibitor
LI: Light Irradiation
Mets: Metastases
MMP-9-R: Matrix Metalloproteinase-9 Responsive
MOF: Metal-Organic Framework
Aclarubicin: Aclacinomycin A
